# Quantitative association between gene expression and blood cell production of individual hematopoietic stem cells in mice

**DOI:** 10.1126/sciadv.adk2132

**Published:** 2024-01-26

**Authors:** Du Jiang, Adnan Y. Chowdhury, Anna Nogalska, Jorge Contreras, Yeachan Lee, Mary Vergel-Rodriguez, Melissa Valenzuela, Rong Lu

**Affiliations:** ^1^ Department of Stem Cell Biology and Regenerative Medicine, Eli and Edythe Broad Center for Regenerative Medicine and Stem Cell Research, Keck School of Medicine, University of Southern California, Los Angeles, CA 90033, USA.; ^2^ Department of Biomedical Engineering, University of Southern California, Los Angeles, CA 90089, USA.; ^3^ Department of Medicine, University of Southern California, Los Angeles, CA 90033, USA.; ^4^ Leonard Davis School of Gerontology, University of Southern California, Los Angeles, CA 90089, USA.

## Abstract

Individual hematopoietic stem cells (HSCs) produce different amounts of blood cells upon transplantation. Taking advantage of the intercellular variation, we developed an experimental and bioinformatic approach to evaluating the quantitative association between gene expression and blood cell production across individual HSCs. We found that most genes associated with blood production exhibit the association only at some levels of blood production. By mapping gene expression with blood production, we identified four distinct patterns of their quantitative association. Some genes consistently correlate with blood production over a range of levels or across all levels, and these genes are found to regulate lymphoid but not myeloid production. Other genes exhibit one or more clear peaks of association. Genes with overlapping peaks are found to be coexpressed in other tissues and share similar molecular functions and regulatory motifs. By dissecting intercellular variations, our findings revealed four quantitative association patterns that reflect distinct dose-response molecular mechanisms modulating the blood cell production of HSCs.

## Teaser

Genome-wide mapping of stem cell gene expression with blood cell production reveals four quantitative association patterns.

## INTRODUCTION

Bone marrow transplantation and its related therapies rely on the blood production of donor hematopoietic stem cells (HSCs) (*1*–*4*). The amounts of blood cells produced by donor HSCs directly determine the efficacy of these therapeutic treatments (*2*, *3*). Previous studies have shown that individual HSCs produce vastly different amounts of blood cells (*5*–*11*) and exhibit different preferences, or “lineage biases,” for the types of blood cells that they produce (*5*–*8*, *10*, *11*). Identifying gene expression signatures associated with distinct quantities of blood cells produced by individual HSCs can provide mechanistic insights into the quantitative modulation of blood reconstitution and can improve the evaluation and optimization of donor cell pools for bone marrow transplantation.

A few recent studies have compared HSCs with high and low amounts of differentiation and observed differences in their epigenetic landscapes (*12*) and gene expression (*13*–*16*). These studies suggest that distinct gene expressions of HSCs underlie their differential lineage output. However, it remains unclear how HSC gene expression underlies varying amounts of blood production. It is possible that genes regulating blood production continuously modulate blood production over a range of blood production levels. However, this constant regulation model is not the only potential molecular regulatory mechanism. For example, blood production may be regulated by genes only when their expression levels increase above certain thresholds. Moreover, many genes function by binding with multiple cofactors and many genomic locations. The expression of these genes likely exhibits a complex association with the lineage output of HSCs. Therefore, conventional differential gene expression analysis comparing cells above and below one particular lineage output level as in previous studies (*13*–*15*) may not capture all relevant genes and in particular may miss those genes that only show correlation within a restricted range of HSC lineage output. In contrast to the previous studies (*13*–*15*), here, we analyzed single-cell gene expression of purified HSCs and measured their lineage output using purified mature blood cell populations from peripheral blood. Moreover, we ranked HSC clones based on their lineage output across multiple mice and identified genes that significantly correlated with the blood cell output at all measured levels across all mice.

Investigating the quantitative associations between single-cell gene expression and single-cell lineage output can not only identify genes associated with blood production at different levels but can also produce insights into the molecular regulatory mechanism of HSC blood production. However, this is difficult due to the large amount of noise in single-cell data that arises from both technical challenges (*17*, *18*) and the stochastic nature of single-cell biology (*19*–*23*). Recent advances in the single-cell transcriptome (*24*–*31*) and clonal tracking analysis (*8*, *11*, *32*–*36*) present exciting opportunities for improving the throughput and quantification of single-cell measurements. Here, we present an experimental system that quantifies single-cell lineage output using clonal tracking data generated by a genetic barcode tracking technique (*8*, *11*, *37*–*39*). This system, in conjunction with “molecular bridges” that connect genetic barcodes and droplet-based single-cell RNA sequencing (*24*, *25*), enables the direct comparison of single-cell lineage output and single-cell gene expression of HSCs. Furthermore, we developed a bioinformatics strategy that can handle noisy single-cell data and map the complex quantitative associations between single-cell gene expression and single-cell lineage output. With these technical advances, we have identified distinct sets of genes whose expression levels in HSCs are significantly associated with specific amounts of blood production. Moreover, we show how these genes are quantitatively associated with blood production across individual HSCs and provide insights into the quantitative modulation of HSC blood reconstitution using the mouse bone marrow transplantation model.

## RESULTS

### Quantifying single-cell differentiation using clonal tracking assays

**Fig. 1.**
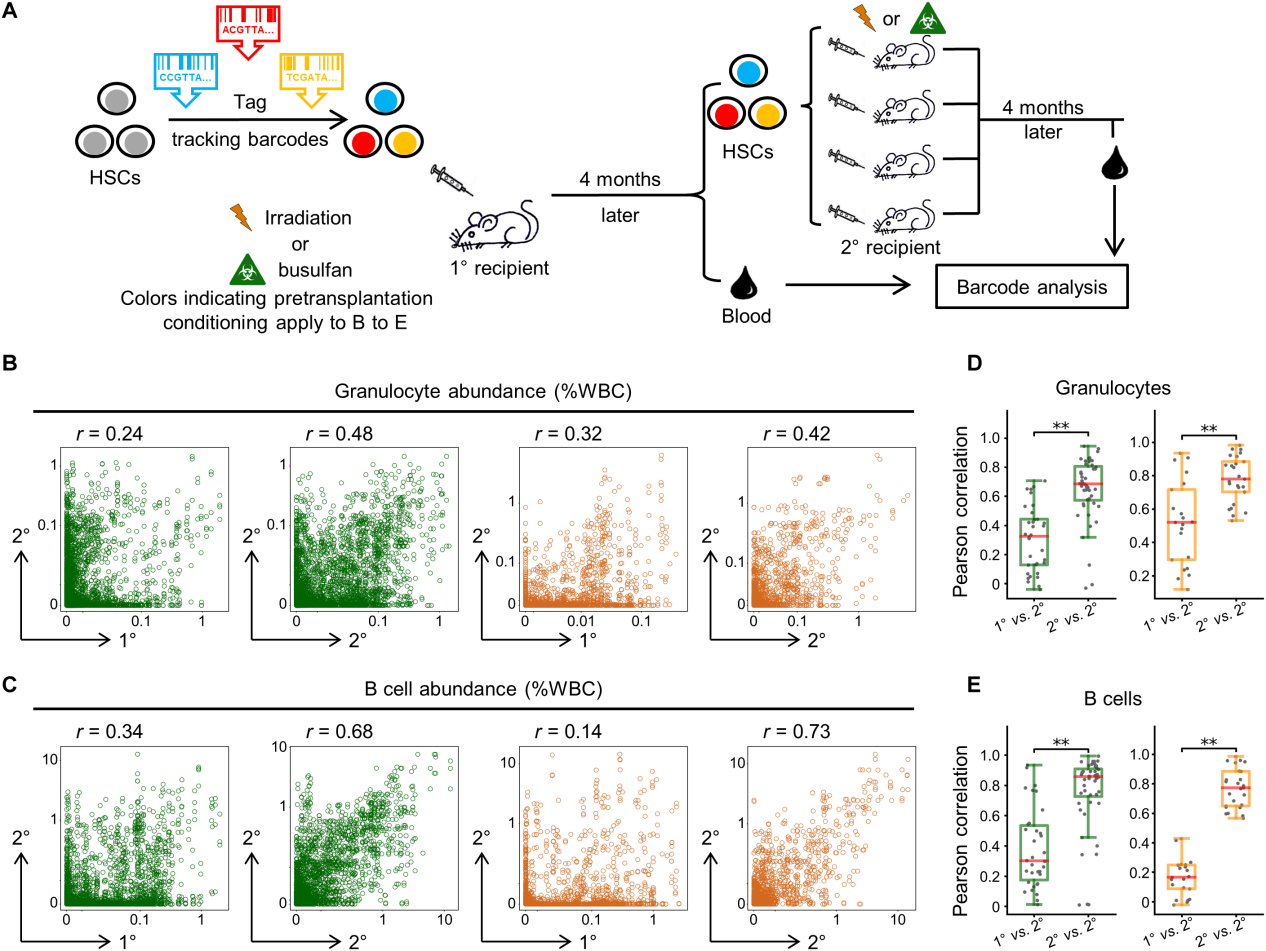
HSCs derived from the same ancestor produce similar amounts of blood cells in different mice. (**A**) Schematic illustration of the one-to-multiple serial transplantation experiment that compares the blood production of HSCs derived from the same ancestor. (**B** and **C**) Comparing the clonal abundance of granulocytes (B) and B cells (C) from primary (1°) and secondary recipients (2°) as well as between two secondary recipients that share a common primary recipient. Each open circle represents an HSC clone, and its position indicates its abundance. Clones from all experimental mice are plotted in each panel. “*r*” depicts Pearson’s correlation coefficient. (**D** and **E**) Pearson’s correlation coefficient for the clonal abundance of granulocytes (D) or B cells (E) between one primary recipient and one secondary recipient (1° versus 2°) and between two secondary recipients (2° versus 2°). Each marker represents a comparison between a pair of mice. The overlaid box plot shows the median and quartiles. WBC, white blood cells. **P < 0.01.

While single-cell RNA sequencing measures the gene expression of individual cells, genetic barcode tracking measures the aggregate lineage output of all HSCs within a clone (*8*, *32*–*35*). To compare data across the single-cell and clonal levels, we first resolved if it is possible to quantify the lineage output of individual HSCs from clonal tracking assays (Fig. 1). Here, HSC lineage output was quantified on the basis of the number of progeny HSCs, granulocytes, and B cells (fig. S1) that were derived from a barcoded HSC [lineage (CD3, CD4, CD8, B220, Gr1, Mac1, Ter119)^−^ cKit^+^ Sca1^+^ Flk2^−^ CD34^−^ CD150^+^]. We use granulocytes and B cells as proxies for studying blood cell production of HSCs in this study because they are the most abundant nucleated myeloid and lymphoid cells, respectively. Taking advantage of the “portable” property of HSCs via transplantation, we compared the amount of in vivo blood production of HSCs derived from the same clone in different mice (Fig. 1A). The data show that although HSC clones changed their blood production between the primary and secondary recipients, HSCs derived from the same ancestor produced consistent amounts of granulocytes and B cells in different secondary recipient mice (Fig. 1, B to E, and fig. S2A). The amounts of self-renewal varied slightly across secondary recipients (fig. S2B), which may be related to the difficulty in collecting all HSCs dispersed in the bone marrow throughout the body even when bone marrow from all major bones was collected as in our experiments (see Materials and Methods). The consistency in blood production was observed in all examined secondary recipient mice, regardless of the use of radiation or chemo treatment as pretransplantation conditioning (Fig. 1, D and E) or the use of cKit-enriched bone marrow cells or fluorescence-activated cell sorting (FACS)–purified HSCs as donor cells in secondary transplantation (fig. S3). The consistency between secondary recipients suggests that the blood production levels are determined by intrinsic cell-autonomous factors that are inherited during HSC self-renewal. The lack of correlation between primary and secondary recipients suggests that HSCs change their blood production to varying degrees upon transplantation, possibly due to the different bone marrow microenvironment that they are exposed to. Together, these data indicate that despite the heterogeneous changes to HSC blood production upon transplantation, the inherent characteristics of HSCs remain the primary factor in determining the blood production quantity, as evidenced by the significant consistency between secondary recipients (Fig. 1, B to E). The consistency between secondary recipients also indicates that the amount of lineage output by individual HSCs can be estimated from clonal-level measurements, assuming individual HSCs within a clone contribute equally.

### Molecular bridges linking clonal tracking and single-cell RNA sequencing

**Fig. 2.**
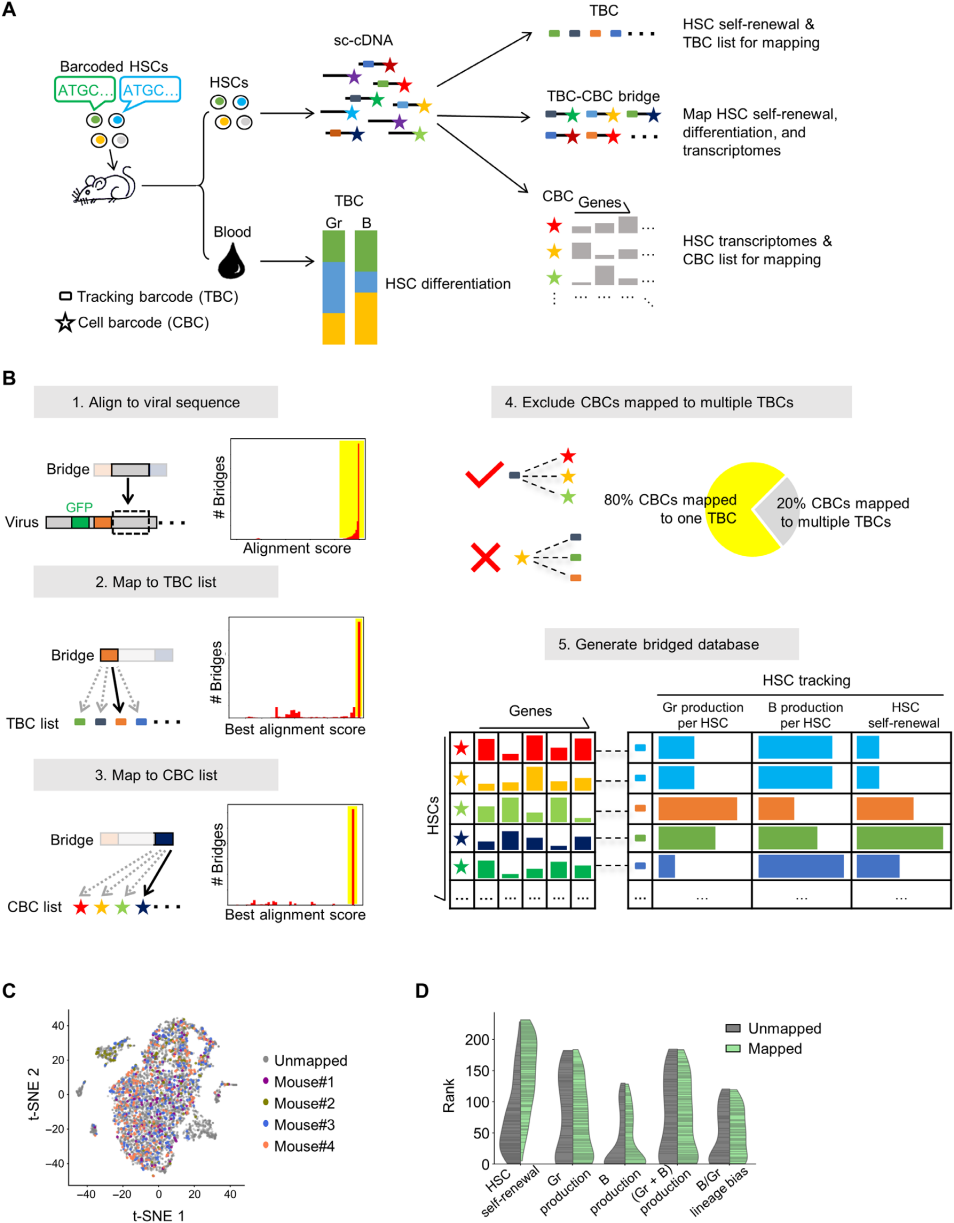
Molecular bridges linking genetic barcode tracking and single-cell RNA sequencing. (**A**) Schematic illustration of the experimental system that simultaneously measures HSC transcriptomes and lineage output in vivo. HSC lineage output is quantified by a tracking barcode (TBC), which is mapped to single-cell transcriptomes through molecular bridges derived from the single-cell cDNA library (sc-cDNA). (**B**) Bioinformatic pipeline for mapping single-cell transcriptomes and single-cell lineage output (see Materials and Methods for details). Yellow bands in the histograms highlight sequencing reads selected for downstream analysis. (**C**) t-SNE (t-distributed stochastic neighbor embedding) plot of HSC single-cell RNA sequencing data comparing HSCs that were mapped to mice through tracking barcodes and HSCs that were not mapped. (**D**) Comparing the lineage output of HSCs that were mapped to single-cell RNA sequencing data and HSCs that were not mapped. The width represents the number of HSC clones at the corresponding lineage output ranks. HSCs, hematopoietic stem cells. Gr, Granulocytes. B, B cells.

We developed an experimental system that simultaneously quantifies in vivo HSC lineage output and their gene expression at the single-cell level in a high-throughput manner (Fig. 2A). The lineage output of hundreds of individual HSCs was quantified by clonal tracking using unique genetic tracking barcodes (*8*, *11*, *37*–*39*). The gene expression of individual HSCs was analyzed by droplet-based single-cell RNA sequencing (*24*, *25*), where the cDNA libraries of individual HSCs were tagged by unique cellular barcodes to allow thousands of individual cells to be simultaneously analyzed. The lineage output and gene expression were measured in the same mouse to eliminate the impact of transplantation on HSC blood production (Fig. 1). To link data from genetic barcode tracking and single-cell RNA sequencing together, we built a library of “molecular bridges,” taking advantage of the presence of transcribed genetic tracking barcodes in the single-cell cDNA libraries (fig. S4). These molecular bridges were generated by polymerase chain reaction (PCR) using primers that specifically amplify the molecules that contain both a genetic tracking barcode and a cellular cDNA barcode (fig. S4A). The molecular bridges were sequenced separately from the cDNA library allowing for improved efficiency and accuracy in mapping barcodes. They constitute a high-sensitivity and high-fidelity directory connecting the two types of barcodes and linking single-cell lineage output with single-cell transcriptomes (Fig. 2B).

In four primary recipient mice 4 months after transplantation, the molecular bridges successfully matched single-cell gene expression and clonal tracking data from 654 HSCs that are affiliated with 156 clones. These numbers were influenced by the proportion of HSCs that were labeled with genetic barcodes during transplantation (Fig. 2A) and by the tracking barcode recovery from the single-cell cDNA library (Fig. 2B). We plotted HSCs from four experimental mice in a UMAP (Uniform Manifold Approximation and Projection) based on their single-cell transcriptome data and found that HSCs from different mice distribute similarly across the UMAP as expected (Fig. 2C). HSCs with different levels of lineage output did not form any cluster on the UMAP. Moreover, HSCs with and without matched datasets exhibited similar transcriptomes (Fig. 2C). We also found that HSCs with and without matched datasets exhibited similar lineage output levels except that HSCs with high amounts of self-renewal were overrepresented among the mapped clones (Fig. 2D). As the molecular bridges were generated by PCR using the single-cell cDNA libraries of HSCs as template, tracking barcodes that marked HSCs with higher amounts of self-renewal were more abundant in the template and therefore more likely to be captured by the molecular bridge library.

### Evaluating the association between gene expression and HSC lineage output at the single-cell level

**Fig. 3.**
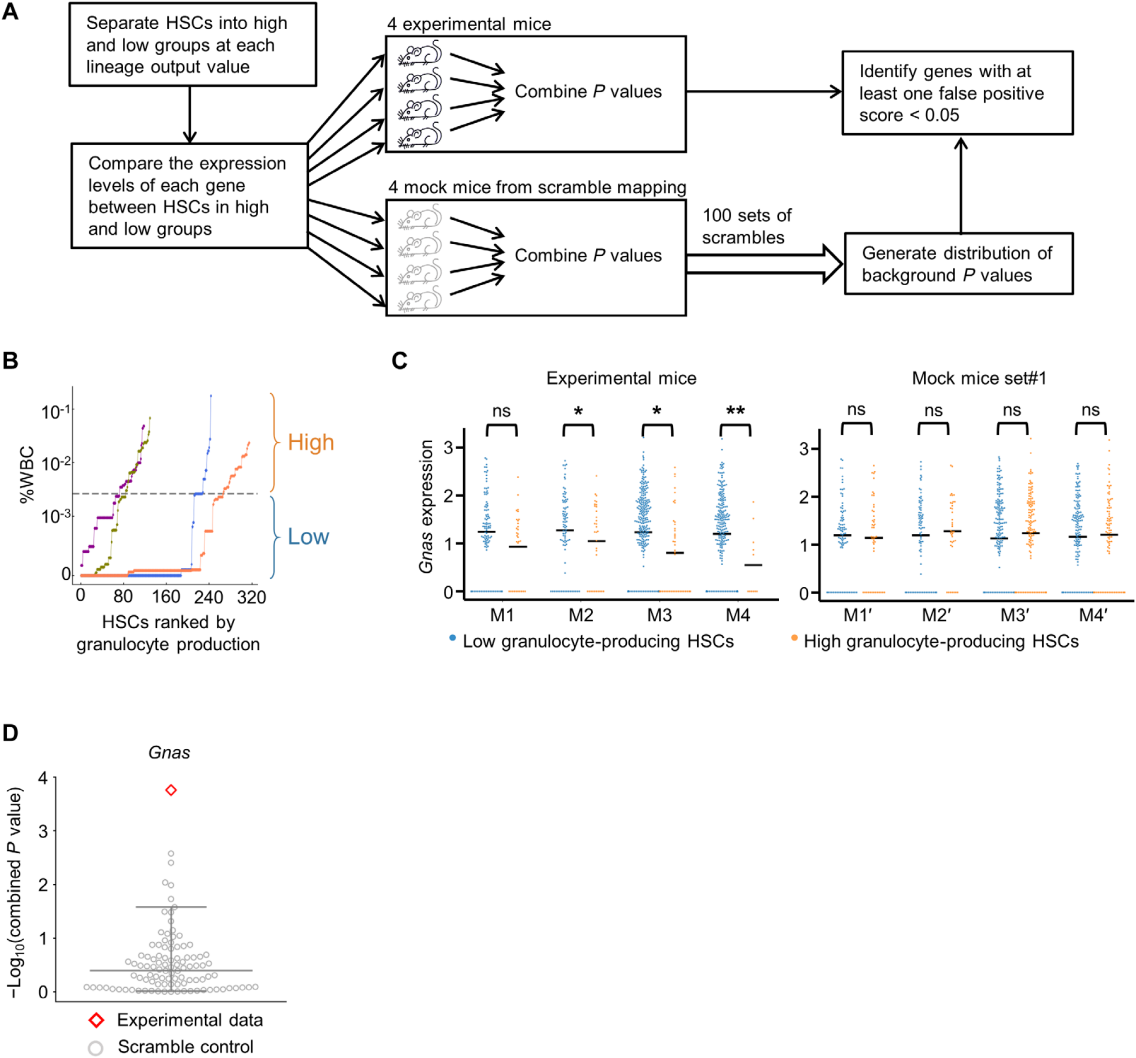
Identifying genes significantly associated with the lineage output of individual HSCs. (**A**) Workflow to identify genes significantly associated with HSC lineage output. (**B**) Individual HSCs are ranked on the basis of their granulocyte production abundance and separated in half into high and low granulocyte-producing groups. Colors denote HSCs from different mice. (**C**) *Gnas* is expressed significantly higher in low granulocyte-producing HSCs compared to high granulocyte-producing HSCs in the experimental mice (M1 to M4) but not in the mock mice generated by scrambling the mapping between tracking barcode data and gene expression data (M1′ to M4′ from one of the 100 scramble sets). (**D**) The combined *P* value for *Gnas* calculated from the experimental data is shown as a red diamond and the combined *P* values for *Gnas* calculated from 100 sets of scramble control data are shown as gray circles. Bars show median and quartiles, 0.05 and 0.95, respectively. (**B** to **D**) The 50% rank of granulocyte production was used as a representative threshold to define low and high granulocyte-producing HSCs. *P < 0.05; **P < 0.01; ns, not significant.

We quantified HSC lineage output in vivo using the following five measurements: HSC self-renewal (HSC abundance), myeloid differentiation (granulocyte abundance), lymphoid differentiation (B cell abundance), differentiation in both lineages (total of granulocyte and B cell abundances), and lineage bias (ratio of B cell to granulocyte abundance). Granulocyte and B cell abundances were quantified as the amounts of granulocytes and B cells that a barcoded HSC produced as a fraction of the entire white blood cell population within a mouse. This allowed for comparisons between different mice. For each measurement of lineage output, we separated HSCs into high and low groups at every detected lineage output value (Fig. 3A). Then, for each mouse, we compared the transcription levels of each gene between the high and low groups (Fig. 3B). Two *P* values were calculated for each gene, representing the probabilities that its expression is associated with either lower or higher amounts of lineage output. Last, we used Fisher’s method to combine the respective *P* values of each gene from different mice. Therefore, the combined *P* values helped identify genes that are significantly associated with a specific HSC lineage output in all experimental mice (Fig. 3C).

To estimate the false positive rate of our gene identification, we compared the experimental data with 100 sets of scrambled data generated by randomly mapping clonal tracking data to gene expression data (see Fig. 3A and Materials and Methods). This was to exclude the possibility that a significant *P* value may arise randomly due to the large number of genes tested. We calculated a false positive score for each *P* value of the experimental data based on whether genes identified by this *P* value can be attained by chance as estimated from the 100 sets of scramble data. This approach was able to identify genes whose differential expression was statistically significant (Fig. 3D).

**Fig. 4.**
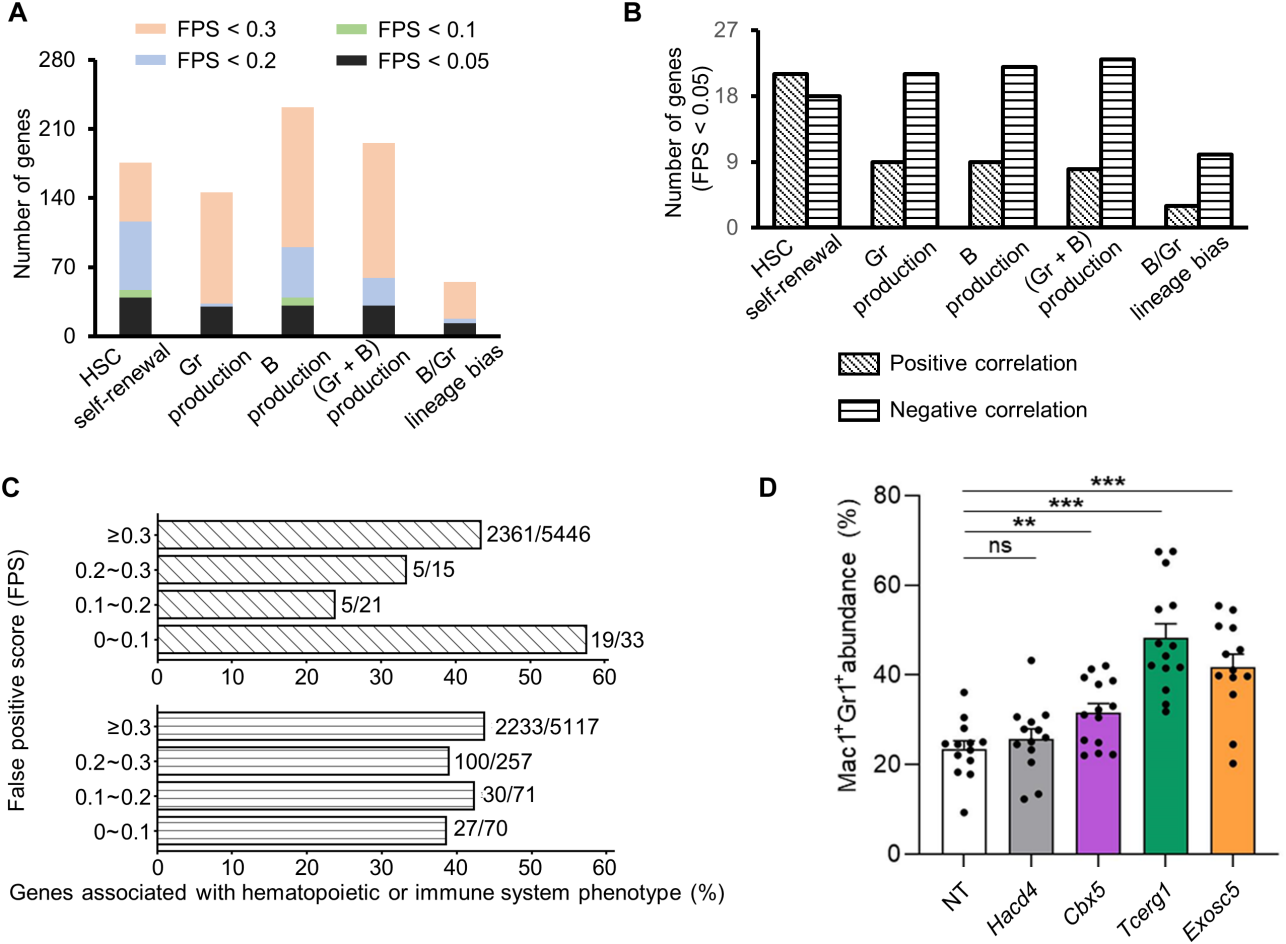
Functional roles of genes significantly associated with the lineage output of individual HSCs. (**A**) Number of genes associated with HSC lineage output at different false positive scores (FPS) using the algorithm outlined in Fig. 3A. (**B**) Number of genes exhibiting positive and negative associations with HSC lineage output. (**C**) Proportion of genes within each FPS range that is associated with hematopoietic or immune system phenotypes on the basis of the MGI mouse phenotype database. This analysis was applied to all genes confidently detected by our HSC single-cell RNA sequencing analyses. Genes were binned on the basis of their positive (top) or negative (bottom) association with HSC lineage output as in (**A** and **B**). The numbers by each bar denote the total number of genes that were annotated with hematopoietic or immune system phenotypes out of those that had any phenotype annotation in the MGI database. *P* = 0.0012 for genes exhibiting positive correlation and *P* = 0.9280 for genes exhibiting negative correlation by the Chi-square test. (**D**) Knockdown of *Cbx5*, *Tcerg1*, and *Exosc5* in HSCs significantly increases the production of Mac1^+^Gr1^+^ granulocytes in vitro. Knockdown of *Hacd4*, which serves as a negative control, did not exert the effect. The experiment was performed twice (*n* = 13 total replicates, except for *Tcerg1* and *Cbx5 n* = 14). Each dot depicts data from one replicate well. Data are shown as means ± SEM. Two-tailed Student’s *t* test. NT, non-targeting control. **P < 0.01; ***P < 0.001.

By reiterating the aforementioned differential gene expression analyses at each detected lineage output value (Fig. 3A), we identified 30 to 40 genes with at least one false positive score less than 0.05 in each HSC lineage output measurement except for the lineage bias measurement, which was deduced from the ratio of two primary measurements (Fig. 4A). Among these genes, more genes exhibited a negative correlation than a positive correlation with blood production (Fig. 4B). In contrast, similar numbers of genes were negatively and positively correlated with HSC self-renewal (Fig. 4B). In addition, fewer genes were identified when data from fewer cells were analyzed (fig. S5). Therefore, a larger sample size can help overcome the background noise. More genes would be identified if more data were available or if the background noise from the experimental measurements could be further reduced. Thus, potentially there exist more extensive associations between gene expression and HSC lineage output at the single-cell level than what we have identified.

### Functional relevance of the identified genes

To comprehensively determine the functional relevance of the identified genes, we searched the Mouse Genome Informatics (MGI) mouse phenotype database. The search result showed that mice carrying mutations in positively correlated genes, but not negatively correlated genes, were significantly more likely to display hematopoietic and immune system phenotypes (Fig. 4C). This is expected as most of the phenotypes were evaluated by mouse models with loss-of-function mutations. Furthermore, many of our identified genes have already been shown to regulate hematopoiesis and are associated with diseases (table S1) (*40*–*67*), consistent with our data. For example, it has been previously shown that overexpression of *Ezh2* in HSCs prevented HSC exhaustion during serial transplantation (*43*). This is consistent with our data that HSCs expressing higher levels of *Ezh2* exhibit greater self-renewal capacities (table S2). In another example, *Sumo* deficiency induces myeloid hyperplasia in zebrafish (*65*) and mice (*66*), supporting our findings that HSCs with reduced *Sumo3* expression produced more granulocytes. Last, overexpression of *Abcg2* in mice has been shown to drive myelodysplastic syndrome, and *Abcg2* expression is particularly high in patients with myelodysplastic syndrome (*56*). This aligns with our findings that HSCs with higher levels of *Abcg2* expression produced more granulocytes.

In addition to the functional relevance shown by previous studies, we performed CRISPR-Cas9–based gene knockout experiments to evaluate the functions of the genes that negatively correlated with granulocyte production (Fig. 4D, fig. S6, and table S3). We used lentiviral vectors to deliver sgRNAs to Cas9-expressing HSCs and induced myeloid differentiation in vitro. Our results suggest that knockout of *Cbx5, Tcerg1*, and *Exosc5* increased granulocyte production, whereas *Hacd4* did not as it is associated with B cell production but not granulocyte production (Fig. 4D). The functional relevance of the identified genes underscores the potential clinical significance of this study.

### Quantitative associations between gene expression and lineage output across individual HSCs

**Fig. 5.**
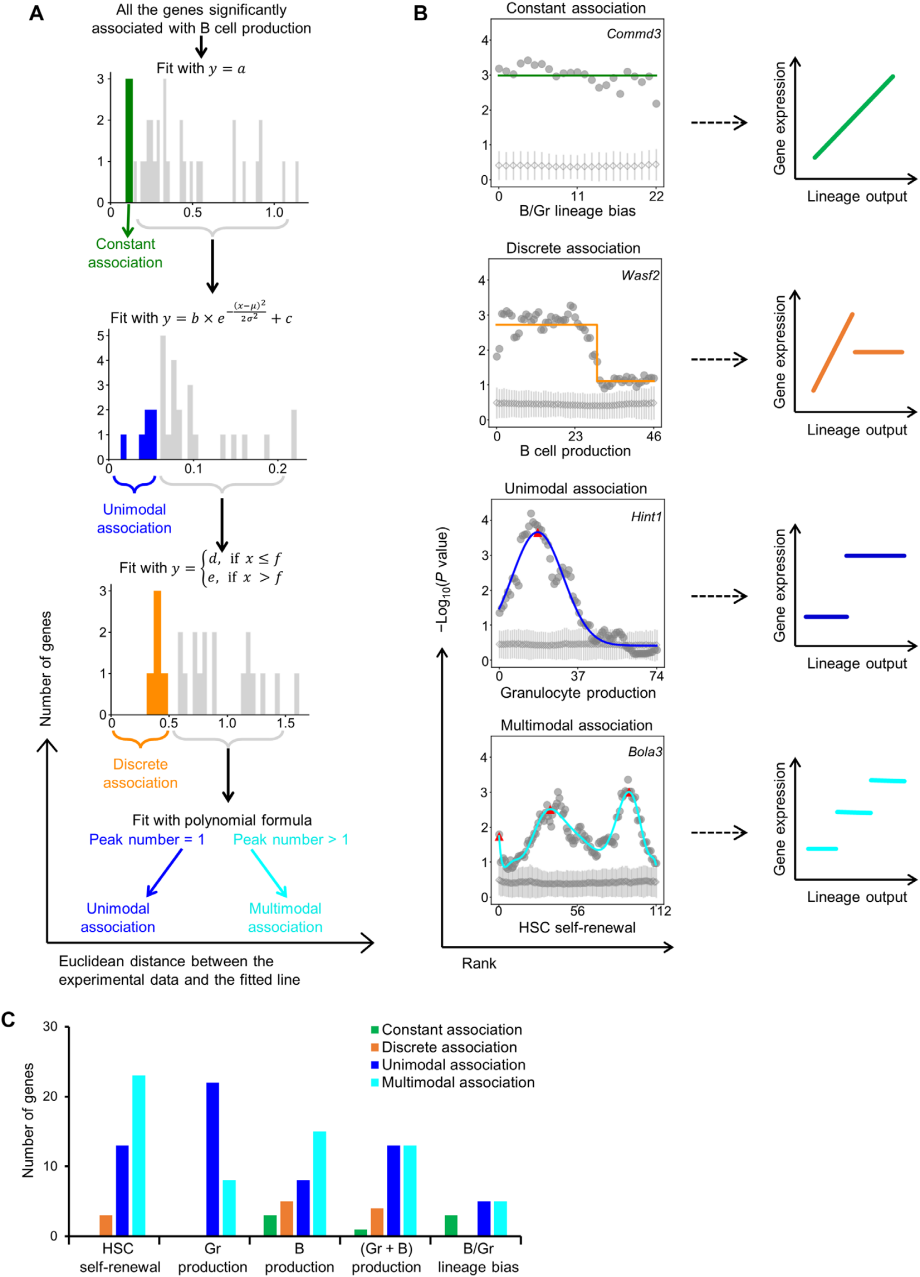
Quantitative associations between HSC gene expression and lineage output at the single-cell level. (**A**) Workflow to classify quantitative association patterns. The quantitative association was determined using two-dimensional data: −log_10_(combined *P* value) derived from the modified differential gene expression analyses (see Fig. 3A) and the rank of HSC B cell production quantity. Histograms show the Euclidean distance between the actual data points and the fitted line. (**B**) Four general association patterns and the corresponding association model. Each graph at the left shows one representative gene that is significantly associated with an HSC lineage output measurement indicated at the bottom. The diagram on the right illustrates the underlying correlation between gene expression and lineage output that is not evident from the raw data (fig. S7). The gene name is shown in the upper right corner. Each gray dot represents a *P* value calculated using one detected HSC lineage output value as the threshold. Each gray diamond represents an averaged *P* value of 100 sets of scramble control data calculated using the corresponding threshold. The gray bar depicts the SDs of all scramble control *P* values. Colored lines show fitted curves. Red triangles denote peak *P* values identified in unimodal and multimodal association patterns. (**C**) The number of significantly associated genes that exhibit a distinct association pattern with HSC lineage output.

Using the aforementioned iterative differential gene expression analysis (Fig. 3A), we mapped the *P* values of each gene across the whole range of a lineage output. Two *P* values were calculated at each lineage output data point to estimate the probabilities that gene expression levels of HSCs with lineage output above this data point were respectively higher or lower compared with those below this data point. This calculation was performed separately for each mouse. Combined *P* values from all experimental mice at each data point were plotted against the rank of the corresponding lineage output. This analysis was only applied to genes that were significantly associated with HSC lineage output with at least one false positive score less than 0.05 (Fig. 4A). We found that each gene’s *P* value changed in a distinctive pattern with the amount of lineage output (Fig. 5). These *P* value patterns show how the association between HSC’s lineage output and gene expression varies at different levels of lineage output. This association dependency is difficult to detect by directly comparing raw gene expression and lineage output data (fig. S7). The *P* values of some genes were consistently high across a wide range and sometimes the entire range of lineage output quantities. However, most gene expression was only correlated with HSC lineage output at specific quantities of lineage output. In some cases, the relationship between the *P* values and lineage output quantity resembled a well-defined normal distribution. In others, the *P* values formed multiple clear peaks at distinct lineage output quantities. On the basis of these observations, we developed an automatic algorithm to classify the patterns of the *P* value variations (Fig. 5A and fig. S8). This algorithm identified four general association patterns between the *P* values and HSC lineage output quantities by sequentially fitting the data to four formulas (Fig. 5B, fig. S9, and table S2). Sequentially fitting the data is necessary because the downstream formulas are more adaptable and flexible than the upstream ones. While this algorithm performs most optimally when the HSC lineage output quantities are presented by rank and the data points are distributed evenly along the *x* axis, the results of the pattern classification are not affected if absolute values of HSC lineage output are used instead (fig. S10). These four general association patterns illustrate how HSC gene expression varies across different amounts of lineage output (Fig. 5B).

**Fig. 6.**
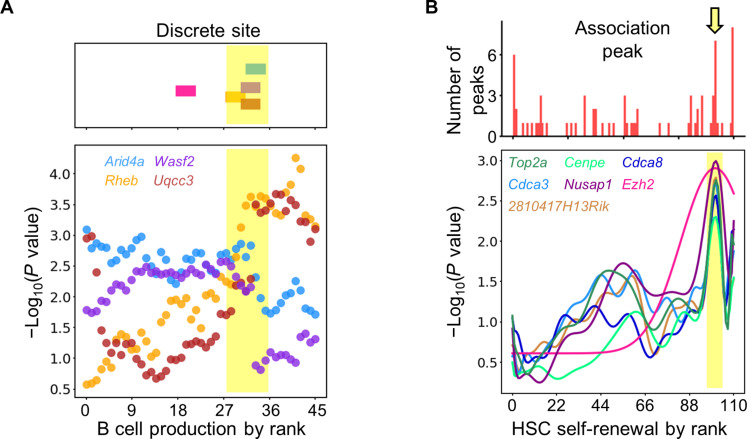
Gene-gene interactions depicted by the quantitative association patterns between gene expression and lineage output of individual HSCs. (**A**) Top: The association between HSC gene expression and B cell production changes abruptly at distinct B cell production quantities illustrated by the position of color bars. Shown are all five genes that exhibit significant discrete associations with B cell production. The yellow band highlights the B cell production levels where the changes to associations of four genes colocalize. Bottom: Quantitative association of the four genes highlighted in yellow at the top. Each dot represents a *P* value calculated using one detected B cell production quantity as the threshold. The color for each gene is consistent between the top and bottom graphs. (**B**) Top: The distribution of *P* value peaks across different HSC self-renewal levels. Shown are all genes that exhibit unimodal or multimodal association with HSC self-renewal. Note that the lowest and highest peaks are composed of genes whose real peaks fall outside of the recorded HSC self-renewal range. Therefore, the data cannot determine if their real peaks colocalize. The yellow arrow highlights the HSC self-renewal level where seven peaks colocalize. Bottom: Smoothed association curves of genes highlighted by the yellow arrow at the top.

1) Constant association: *P* values are significant and steady (Fig. 5B), indicating that these genes are significantly differentially expressed across all levels of lineage output. This association pattern implies a dose-response relationship where gene expression continuously increases or decreases with the lineage output. Genes exhibiting this association pattern, such as *Commd3* (Fig. 5B), can be used to alter the blood production of all HSCs regardless of their blood production status.

2) Discrete association: *P* values shift abruptly and are generally constant outside of the few transition points (Fig. 5B). This pattern indicates that the association between gene expression and HSC lineage output is limited to a specific range of lineage output levels. For example, the expression of *Wasf2* is associated with B cell production only when B cell production levels are low (Fig. 5B). When B cell production levels are high, *Wasf2*’s expression is no longer associated with B cell production. If these genes regulate HSC lineage output, then alternative genes and mechanisms must exist to carry out the regulation outside of the association ranges. For example, we found that *Rheb* and *Uqcc3* exhibit correlation specifically at high levels of B cell production when *Wasf2* is uncorrelated (Fig. 6A).

3) Unimodal association: This *P* value association pattern generally resembles a normal distribution with one clear peak, indicating that the gene was most significantly differentially expressed at one specific level of lineage output (Fig. 5B). This association pattern can be explained by a simple model where the association peak delineates the separation between HSCs expressing the gene at high and low states. Alteration in lineage output is associated with the transition between the two gene expression states and not with changes in gene expression within either state. The position of the association peak in effect denotes the lineage output level where this gene becomes significantly contributing or relating to lineage output. Mechanistically, this association pattern resembles an on/off toggle switch. Biologically, this pattern may be the result of cooperative binding between different molecules that requires a certain molecular concentration conferred by a particular gene expression level. If genes exhibiting unimodal association, such as *Hint1* (Fig. 5B), are essential for their respective HSC lineage output, then their expression levels may need to reach a critical threshold to alter lineage output.

4) Multimodal association: *P* value distribution forms multiple peaks (Fig. 5B). These genes are most significantly differentially expressed at several distinct quantities of HSC lineage output. Similar to the unimodal association pattern, the multimodal association pattern suggests that there are multiple lineage output quantities where gene expression changes are significantly associated with lineage output. This may be the result of a gene having multiple binding partners in regulating lineage output. The positions of the multiple association peaks in effect denote the lineage output levels where each binding partner becomes significantly contributing or relating to lineage output.

### Prevalence of the quantitative association patterns

Unimodal and multimodal associations are the most common quantitative association patterns among the genes significantly associated with HSC lineage output that we identified (Fig. 5C). These association patterns suggest that gene expression must reach distinct thresholds to trigger quantitative changes to HSC lineage output and that cooperative binding between different molecules is extensively involved in regulating HSC lineage output.

On the other hand, no constant association pattern was found to govern HSC self-renewal and granulocyte production (Fig. 5C). The discrete association pattern was also absent from granulocyte production and was rarely seen to modulate HSC self-renewal (Fig. 5C). Constant association and discrete association patterns both suggest that gene expression continuously increases or decreases with the quantity of lineage output. These data indicate that this type of dose-response mechanism may not be involved in regulating HSC self-renewal and myeloid production, although it may play a role in regulating lymphoid production. The different quantitative association patterns between granulocytes and B cells may be related to differences in their regeneration dynamics, as granulocytes typically have shorter lifespans and are replenished more rapidly compared to B cells.

### Gene-gene interactions revealed by the quantitative association patterns

Genes exhibiting similar association patterns often share common transition points in their *P* values (Fig. 6, A and B). The transition points signify changes in the association between HSC gene expression and lineage output at distinct lineage output levels. For example, the transition points of the discrete association pattern depict the lineage output levels where the corresponding genes alter their association with lineage output. Out of the five genes that were significantly associated with B cell production and exhibited the discrete association pattern, four showed abrupt increases or decreases in *P* values at similar quantities of B cell production (Fig. 6A). Among the four genes, *Arid4a* and *Wasf2* were significantly associated with B cell production below this quantity; *Rheb* and *Uqcc3* were significantly associated with B cell production above this quantity. These four genes may be involved in alternative molecular programs that regulate HSCs in producing B cells at either high or low levels.

The association peaks in unimodal and multimodal association patterns denote the lineage output levels where corresponding genes become significantly contributing or relating to lineage output. Colocalized peaks suggest that different binding events act at the same lineage output level, possibly by influencing the same molecular machinery or pathway. For example, seven genes associated with HSC self-renewal displayed association peaks around the same high level of self-renewal (Fig. 6B). It indicates that these genes may be collectively involved in the same molecular machinery or pathway underlying HSC self-renewal.

### Genes with overlapping association peaks exhibit significant functional and molecular connections

**Fig. 7.**
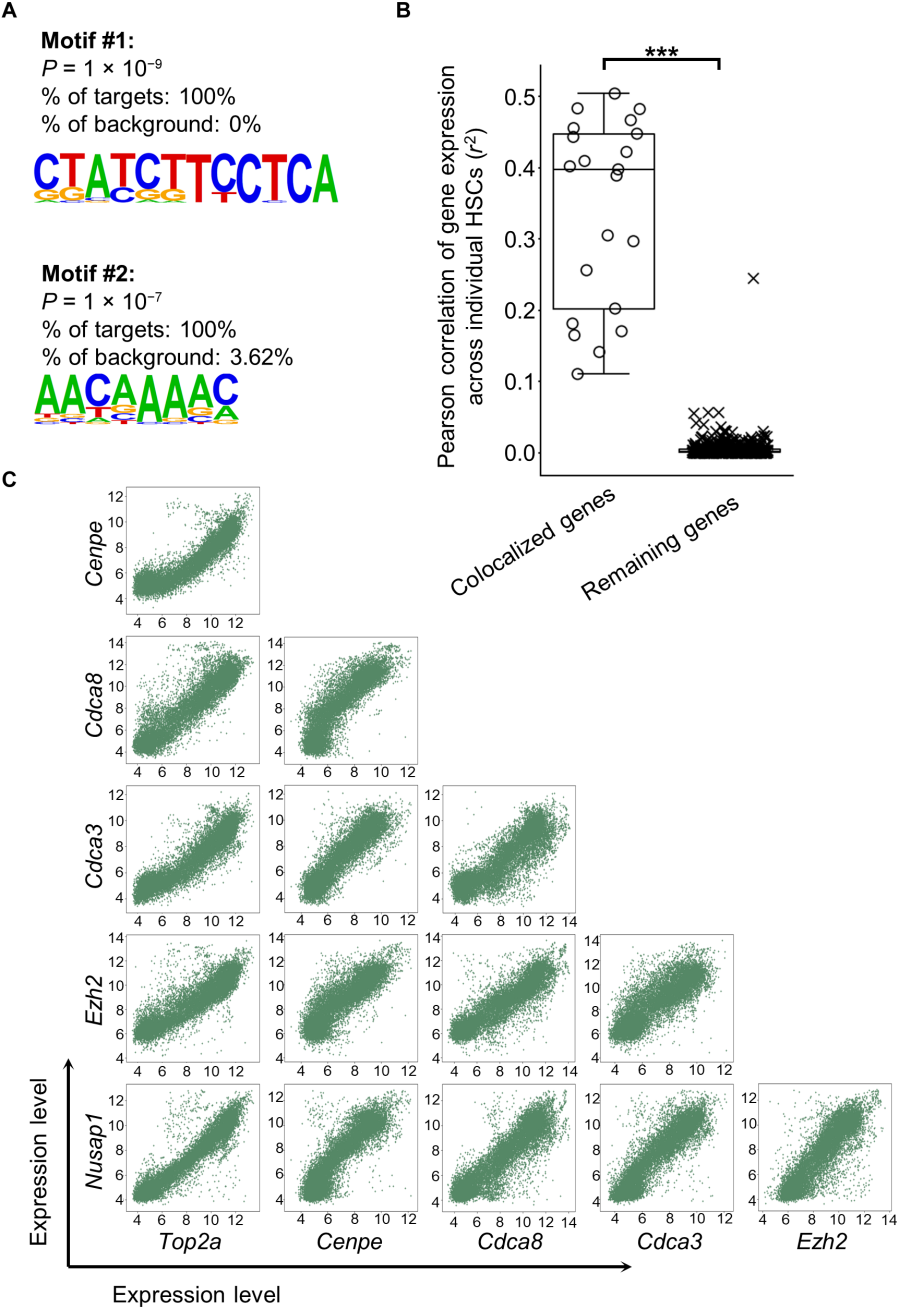
Genes with overlapping association peaks exhibit significant connections. (**A**) Common motifs shared across the genes highlighted in yellow in (Fig. 6B). Analysis was performed by Homer48 (v4.11.1). The remaining genes exhibiting unimodal or multimodal association with HSC self-renewal were used as background in the motif identification. (**B**) Correlation in gene expression across individual HSCs between genes exhibiting unimodal or multimodal association with HSC self-renewal. Each circle represents a comparison of two genes highlighted in yellow in (Fig. 6B). Each “×” mark represents a comparison of the remaining genes exhibiting unimodal or multimodal association with HSC self-renewal. *r*^2^ was used to compare both positive and negative correlations. The overlaid box plot shows the median and quartiles. ***P < 0.001. (**C**) Pairwise comparison of the genes highlighted in yellow in (Fig. 6B) for their transcription levels as measured by mRNA microarrays across different mouse tissues (*44*) (http://hegemon.ucsd.edu/Tools/explore.php?key=global, Dataset “Mouse 430 2.0”). One of the seven highlighted genes (2810417H13Rik) was not found in this dataset. Each dot represents data from one microarray analysis. The position of the dot shows the normalized expression levels of the corresponding genes.

Because of the limitations of current technology, we cannot manipulate the levels of gene expression or lineage output in vivo. Therefore, to validate the quantitative association patterns that we have identified, we studied the functional and molecular connections between genes that exhibit overlapping association peaks (Fig. 6B). Among the seven genes that exhibit overlapping association peaks at high levels of HSC self-renewal, we found that six genes are known to play roles in cell cycle, and the remaining one is a DNA topoisomerase (table S4). HSC clones displaying high or low levels of self-renewal exhibit similar cell cycle state distributions (fig. S11). Moreover, a previous study has shown that six of the seven genes are significantly up-regulated in spermatogonial stem cells during regeneration as compared to a homeostatic state (*68*). Our analyses further reveal that these seven genes also share common regulatory motifs at their promoter regions in mice and humans (Fig. 7A and figs. S12 and S13A). In addition, the expression of these genes is correlated with each other across individual HSCs (Fig. 7B). The correlation of gene expression is also identified by comparing all published mRNA microarray data from different mouse tissues (Fig. 7C) and from different human tissues (fig. S13B) (*69*). The significant coexpression across tissues and organisms, similar regulatory motifs, and molecular functions for genes with colocalized association peaks confirm the accuracy of our identified quantitative association patterns.

## DISCUSSION

In this study, we showed that most genes that are associated with HSC blood production are associated only at specific levels of blood production. Moreover, we presented a framework for measuring the quantitative association between single-cell gene expression and single-cell lineage output by mapping each gene’s correlation with blood production across the entire range of blood production levels (Fig. 5 and fig. S9). The advantage of this approach is demonstrated by its ability to overcome the noise associated with comparing two single-cell measurements. Our other attempts at modeling and comparing the single-cell datasets failed to generate any meaningful quantitative connection between gene expression and lineage output. In particular, attempts to compare cells based on gene expression were unable to reveal any significant difference in lineage output, indicating that single-cell gene expression measurements may contain more noise, likely both technical noise and biological noise, than single-cell lineage output measurements. Our presented approach uses biological replicates to increase the statistical power (Fig. 3A) and uses the association *P* values, instead of the raw gene expression data, to identify the quantitative association patterns (Fig. 5 and fig. S7). Current technologies do not allow for quantitative manipulation of gene expression or lineage output in vivo. If future technological advancements enable precise fine-tuning of gene expression in specific subsets of HSCs that exhibit distinct levels of blood cell production, then it could provide an opportunity to validate or challenge the functional relevance of the identified quantitative association patterns. Nonetheless, we provided orthogonal supporting evidence for the quantitative association patterns by demonstrating that genes with similar association patterns have similar molecular functions and regulatory motifs and are also coexpressed in other tissues of mice and humans (Fig. 7 and fig. S13). We also validated the association of selected genes with granulocyte production in vitro (Fig. 4D).

Identification of the four general association patterns suggests that HSCs producing different amounts of blood cells are regulated by distinct sets of molecular machinery. Genes that share a common transition point may be closely related in the molecular regulatory network modulating the lineage output of HSCs (Figs. 6 and 7). These association patterns reflect elemental regulatory modes across cellular and molecular levels and provide an original perspective for deciphering and modeling complex gene regulatory networks. Moreover, the quantitative association patterns provide evidence that intercellular variations in gene expression reflect active management of in vivo HSC lineage output through distinct dose-response mechanisms. In contrast, previous studies of single-cell organisms suggest that intercellular variations in gene expression arise from stochastic transcriptional events and do not have any functional consequence (*19*–*23*). In multicellular organisms, multiple cells performing an identical function must be coordinated to ensure a proper level of biological function overall within an organism. Part of this coordination may rely on fine-tuning the function of each cell. Our data suggest that this fine-tuning may be carried out in multiple ways through distinct dose-response mechanisms. Dose-dependent cell fate regulation has been shown for a few genes at the cell population level (*70*–*72*). Here, our single-cell transcriptomic study offers original and more comprehensive insights into the dose-dependent regulatory mechanism of cell fate.

Our quantitative association analyses relate specific HSC genes to distinct levels of lineage output (Fig. 5, fig. S9, and table S2). These results can help evaluate and optimize donor HSC pools for bone marrow transplantation. In particular, optimal donor HSCs could be those that express genes associated with high blood production levels as well as balanced blood production. Understanding the nature of these molecular and cellular associations can also enable better control of blood production in biomedical applications. For instance, genes exhibiting the constant association pattern can be good therapeutic targets for controlling blood production as they can affect all HSCs regardless of their blood production status. On the other hand, genes specifically associated with high levels of HSC self-renewal can be good therapeutic targets for tuning down cellular proliferation without completely blocking it. Cancer treatments may target this class of genes to inhibit the excessive proliferation of malignant cells without completely blocking the proliferation of normal cells.

Our approach is based on single-cell transcriptome measurements of HSCs. Therefore, this study did not consider other influencing factors for the lineage output of HSCs, such as their chromatin state and microenvironment in the bone marrow. In addition, our study uses granulocytes and B cells as measurements of HSC blood production, and it is possible that the findings may not apply to the other blood cell types. We did not use T cell measurement in this study because we have found that after unconditioned transplantation, granulocytes and B cells exhibit a linear clonal correlation with HSCs, while T cells do not (*11*). This is likely due to the clonal selection that takes place during the maturation of T cells in the thymus. Nonetheless, our study provides a framework for comparing single-cell gene expression and single-cell lineage output quantitatively. We identified genes that are expressed in relation to the blood production of individual HSCs at distinct levels (table S2). Many of these genes have been previously linked to hematopoiesis (Fig. 4C and table S1). Our study also revealed potential regulatory genes (Fig. 4D).

Our experimental system offers three major technical advances over other existing techniques that attempt to connect molecular profiles with lineage output at the single-cell level (*13*–*15*). First, we achieved single-cell–to–single-cell mapping between gene expression and lineage output by quantifying single-cell lineage output using clonal tracking data based on our finding that HSCs derived from the same clone produce similar numbers of blood cells (Fig. 1). Our single-cell–level analyses of clonal-level measurements represent an improvement over other methods that could only connect single-cell gene expression profiles to the clonal-level differentiation of many individual cells (*13*, *14*). Second, we generated a library of “molecular bridges” to connect genetic barcode tracking and droplet-based single-cell RNA sequencing with enhanced efficiency and accuracy (Fig. 2). The unique and versatile design of our molecular bridges allows for their broad application to any cellular function assay where tracking barcodes are transcribed. They are neither reliant upon any specific transgenic mice nor locked to a particular tracking barcode design as in other experimental systems (*13*, *14*). Third, we developed a bioinformatics framework that can identify complex quantitative associations between single-cell gene expression and single-cell lineage output (Fig. 3) in contrast to the conventional differential gene expression analyses used in previous studies (*13*–*15*). Our experimental and bioinformatic approach is easily adaptable to a wide variety of biological systems where cells can be tracked by transcribed genetic barcodes. By quantitatively dissecting intercellular variations, this approach allows for identifying regulators and regulatory mechanisms that mediate cell fate determination at the single-cell level.

## MATERIALS AND METHODS

### Experimental design

#### Mice

The primary donor mice used in all the experiments were 8- to 12-week-old B6 (C57BL/6 J, CD45.2^+^, The Jackson Laboratory, #000664) or F1 (CD45.2^+^/CD45.1^+^, crossbreed B6 and BLY mice in-house) mice. The recipient mice were 8- to 12-week-old BLY (B6.SJL-Ptprca Pepcb/BoyJ, CD45.1^+^, The Jackson Laboratory, #002014) mice. Cells obtained from the Cas9-expressing mice [B6J.129(Cg)-Gt(ROSA)26Sortm1.1(CAG-cas9*,-EGFP)Fezh/J, The Jackson Laboratory, #026179] were used for the in vitro myeloid differentiation assay. All animal procedures were approved by the Institutional Animal Care and Use Committee.

#### HSC isolation and transplantation

In the primary transplantation experiments, HSCs [lineage (CD3, CD4, CD8, B220, Gr1, Mac1, Ter119)−/cKit^+^/Sca1^+^/Flk2^−^/CD34^−^/CD150^+^] were obtained from the crushed bones of donor mice and isolated using FACS with the FACSAria II (BD Biosciences, San Jose, CA) after enrichment using CD117 (cKit) microbeads (AutoMACS, Miltenyi Biotec, #130-091-224). Before primary transplantation, HSCs were infected with lentivirus carrying barcodes and green fluorescent protein (GFP) for 15 hours. HSC clonal labeling was carried out as previously described (*8*, *37*). Helper cells that were co-transplanted alongside HSCs consisted of F1 or BLY whole bone marrow cells. Before transplantation, recipient mice were treated with one of the following two conditions: (i) irradiation with 950 cGy immediately before transplantation (nine primary recipients and their corresponding secondary recipients) or (ii) intraperitoneal injection with busulfan (Sigma-Aldrich, #150606) at a dose of 50 mg per kg body weight 24 hours before transplantation (*73*) (seven primary recipients and their corresponding secondary recipients). Barcoded donor HSCs (irradiation group: 2500 HSCs for three mice and 7000 HSCs for five mice; busulfan group: 2500 HSCs for three mice and 4000 HSCs for four mice) and 250,000 helper bone marrow cells were transplanted into each recipient in four batches to ensure biological reproducibility. Donor cell dose did not influence any results reported in this study, so data from mice transplanted with different HSC doses were combined in the same group. During the secondary transplantation, CD117^−^ microbead-enriched bone marrow cells (Fig. 1) or FACS-purified HSCs (fig. S3) from each primary recipient were equally divided into four portions and transplanted into secondary recipients. A total of 250,000 whole bone marrow cells (helper cells) were also transplanted into the secondary recipients who received FACS-purified HSCs. Both primary and secondary transplantations were performed via retro-orbital injection. Ten months after secondary transplantation, mice were euthanized, and their HSCs were sorted as described above. At the end time point, transplantation quality was evaluated by FACS. Donor chimerism of all recipient mice was 96.6 ± 3.9% in granulocytes and 94.6 ± 2.4% in HSCs; GFP% was 63.9 ± 12.7% in granulocytes and 49.2 ± 8.5% in HSCs.

#### Blood sample collection and FACS analysis

Blood samples were collected into phosphate-buffered saline (PBS) containing 10 mM EDTA via a small transverse cut in the tail vein of alive mice. At the end-time point, blood was collected using transcardial perfusion with 10 mM EDTA PBS. After blood collection, 2% dextran was added to eliminate red blood cells, and the remaining blood cells were incubated with ammonium-chloride-potassium lysis buffer on ice for 5 min to remove residual red blood cells. After a 30-min antibody incubation at 4°C, samples were resuspended in propidium iodide solution [1:5000 in PBS with 2% fetal bovine serum (FBS)]. Cells were sorted using FACSAria II and separated into granulocytes and B cells. Flow cytometry data were analyzed using BD FACSDiva software version 8.0. Antibodies were obtained from eBioscience and BioLegend, as previously described (*38*, *39*). Donor cells were sorted on the basis of the CD45 marker. The following cell-surface markers were used to sort the different blood cell types:

Granulocytes: CD4^−^/CD8^−^/B220^−^CD19^−^/Mac1^+^/Gr1^+^.

B cells: CD4^−^/CD8^−^Gr1^−^/Mac1^−^/B220^+^/CD19^+^.

#### Tracking barcode extraction

Genomic DNA was extracted from sorted hematopoietic cells and amplified using Phusion PCR Master Mix (Thermo Fisher Scientific, #F-531 L). PCR reactions were halted once they had progressed halfway through the exponential phase. The PCR product was purified and analyzed using an Illumina NextSeq 500 at the USC Epigenome Center. Sequencing data were analyzed, as previously described (*37*–*39*). The lentiviral vector that delivers genetic barcodes also conveys GFP expression that marks barcoded cells.

#### Single-cell RNA sequencing

HSCs from four recipient mice (two males, two females) were FACS-sorted on the same day and processed in the same 10xChromium chip. Single HSC suspensions were washed with ice-cold 0.04% BSA in PBS, loaded onto 3′ library chips, and processed according to the manufacturer’s protocol for the Chromium Single Cell 3’ Library (10X Genomics, V2) with minor modifications as follows. A total of 5000 to 9000 HSCs from each mouse were loaded into each reaction channel. The resulting cDNAs were then amplified per the manufacturer’s recommendation with one additional cycle. After cDNA amplification, half of the amplified cDNA was used for the downstream fragmentation, adaptor ligation, and sample index PCR. The other half of the cDNA was used to amplify “molecular bridges” that contain the genetic tracking barcodes and the Chromium cellular barcodes (see below). The former indexed cDNA libraries were first sequenced on an Illumina NextSeq 500 aiming at a coverage of 5000 raw reads per cell to estimate the cell numbers, before sequencing deeper on an Illumina HiSeq 4000 aiming at a coverage of 50,000 raw reads per cell (paired-end; read1: 26 cycles; i7 index: 8 cycles; read 2: 98 cycles). The sequencing results were processed using the Cell Ranger pipeline (10x Genomics, v 2.1.0) for cellular barcode assignment and unique molecule identifier (UMI) quantification. A total of 7604 cells were identified, with an average sequencing depth of 52,982 reads per cell. Cells with more than 5% UMIs mapped to mitochondrial genes were excluded. This filter returned 6899 cells for further analysis. Only genes with at least three UMIs in at least 5% of cells were used for downstream analyses. Expression values for gene *i* in cell *j* were calculated by dividing UMI count values for gene *i* by the sum of the UMI counts in cell *j*, and then multiplying by 10,000 to create TPM-like values, and lastly transforming to log_2_(TPM + 1).

#### Generating and mapping molecular bridges

To specifically amplify molecular bridges containing both a clonal tracking barcode and a cell barcode, we performed PCR using the single-cell cDNA library as a template and a single primer (5′- sample index-ACACTCTTTCCCTACACGAC) (fig. S4A). The PCR products, larger than 1.5 kb long (fig. S4A), were purified from a band after agarose gel electrophoresis (Zymo Research, #11-300C) (fig. S4B), and then sequenced using the PacBio Sequel sequencer (Pacific Biosciences, v2.1).

Raw PacBio sequencing data were analyzed using circular consensus sequences application of SMRT Analysis software (Pacific Biosciences, SMRT Link Version 5.1.0) with default parameters. The resulting “fasta” files were processed by a customized Python script (Fig. 2B). We first aligned the molecular bridges to the viral sequence, the main part of the molecular bridges. Then, the well-aligned molecular bridges were compared to the genetic tracking barcode list and the Chromium cellular barcode list generated from the same HSC population (Fig. 2A). In addition to one-to-one mapping between tracking barcodes and cellular barcodes, we allow one tracking barcode to map to multiple cellular barcodes, as each tracking barcode represents one HSC clone that may consist of multiple HSCs upon self-renewal. Around 20% of cellular barcodes were mapped to multiple tracking barcodes, which we discarded from the downstream analysis. They may have arisen from multiple viral infections during genetic barcoding labeling, or from multiple cells captured in the same droplet during single-cell cDNA library generation. Both are random processes and difficult to avoid.

#### In vitro myeloid differentiation assay

FACS-sorted HSCs [lineage (CD3, CD4, CD8, B220, Gr1, Mac1, Ter119)−/cKit^+^/Sca1^+^/CD34^−^/CD150^+^] from Cas9-expressing mice were plated in Improved Minimum Essential Medium (IMEM) with 10% FBS and 1% penicillin/streptomycin and transduced with lentivirus carrying a mix of three different single-guide RNAs (sgRNAs) targeting each of the selected genes (*Tcerg1*, *Exosc5*, and *Cbx1*). Non-genome–targeting sgRNAs and sgRNAs targeting *Hacd4* were used as negative controls. sgRNAs were designed and cloned as previously described (table S3) (*37*, *74*). The transduced cells were subsequently cultured in the media supplemented with mSCF (50 ng/ml), mIL-3 (10 ng/ml), hIL-6 (10 ng/ml), and hEPO (10 ng/ml) to facilitate their myeloid differentiation (*75*). After 8 days of culture, the abundance of differentiated granulocytes (Mac1^+^ Gr1^+^ cells) among all transduced (RFP^+^) cells was analyzed by FACS (Fig. 4D and fig. S6). The transduced cells (RFP^+^) were also sorted and used for on-target editing verification by Sanger sequencing followed by indel analysis using the Inference of CRISPR Edits (ICE) from Synthego (www.synthego.com/products/bioinformatics/crispr-analysis) (*76*)(table S3).

### Statistical analysis

#### Quantification of HSC lineage output

While we were able to confidently quantify HSC blood production by thoroughly harvesting the vast majority of the peripheral blood cells through perfusion, our measurement of HSC self-renewal is influenced by the accessibility to HSCs in the bone marrow. We harvested bone marrow from all of the major bones including the spine, legs, and arms. The specific calculations of clonal abundance are as follows:

$$\begin{array}{c} \text{Clonal abundance in the blood}\;\left(\%\text{WBC}\right)\\ =\left(\text{cell type}\;\%\;\text{white blood cells}\right)\times \left(\text{donor}\;\%\right)\times \left(\text{GFP}\%\;\text{among donor cells}\right)\\ \times \left(\text{read number of}\;\text{one}\;\text{barcode}\right)/\left(\text{total reads of all barcodes}\right) \end{array}$$

$$\begin{array}{c} \text{Clonal abundance of HSCs}\;\left(\%\;\text{barcoded HSCs}\right)\\ =\left(\text{read number of}\;\text{one}\;\text{barcode}\right)\times 100\%/\left(\text{total reads of}\;\text{all}\;\text{barcodes}\right) \end{array}$$

Clones whose contributions to white blood cells were lower than 0.001% were excluded from all analyses. To ensure accurate quantification of lineage bias, clones whose abundances were less than 1% of the most abundant clones from both lineages were excluded from the lineage bias analysis only. HSC self-renewal was quantified as the clonal abundance of HSCs that share an identical tracking barcode among all harvested HSCs with barcodes. HSC differentiation was quantified as the clonal abundance of all white blood cells in the peripheral blood divided by the clonal abundance in HSCs (Fig. 2A).

#### Quantification of HSC lineage bias

The calculation of lineage bias is as follows:

If clonal abundance in granulocytes = 0 and clonal abundance in B cells > 0, lineage bias level = 1

If clonal abundance in granulocytes > 0 and clonal abundance in B cells = 0, lineage bias level = − 1

If clonal abundance in granulocytes > 0 and clonal abundance in B cells > 0,

$$\text{lineage bias level}=\frac{\text{arctan}\frac{\left(\text{clonal abundance in}\;B\;\text{cells}\right)/\left(\text{total}\;B\;\text{cells}\%\text{white blood cells}\right)}{\left(\text{clonal abundance in granulocytes}\right)/\text{total granulocyts}\%\text{white blood cells})}-\pi /4}{\frac{\pi }{4}}$$

#### Identifying differentially expressed genes

To identify genes whose expression levels are significantly associated with variations in HSC lineage output, we adopted the conventional approach of comparing the gene expression of HSCs with high and low lineage outputs, and we made two modifications as follows.

First, we performed the high versus low comparison at all valid thresholds that separate HSCs into the “high” and “low” groups. A valid threshold is defined as a measured lineage output value where the number of HSCs above or below this value is more than four in each mouse (Fig. 3A).

Second, we use scramble control to estimate the background distribution of *P* values and calculate the false positive score that depicts the possibility that a given *P* value can be generated by chance. We generated 100 sets of scramble control data. In each set, four “mock” mice were created by randomly mapping genetic tracking barcode data to gene expression data (Fig. 3A). This random mapping allowed for changes in the number of cellular barcodes that mapped to one tracking barcode.

For the scramble control and experimental data, we performed the following analysis at each valid threshold:

1) For each gene, calculate two *P* values using the one-sided Mann-Whitney test by comparing the gene expression levels of HSCs in the “high” and “low” groups. These two *P* values represent the probability that this gene is expressed at a higher level in HSCs with higher or lower lineage outputs (two “directions”).

2) For each gene, combine the *P* values of the same direction from all replicate mice in the experimental group or in each set of the control group using Fisher’s method.

3) For each gene in each direction, count the number of genes (*N*) whose *P* values were equal to or smaller than this gene in the experimental group or in each set of the control group.

4) Calculate a false positive score for each gene in each direction, which was defined as *N* of the control group (median of the 100 sets) divided by *N* of the experimental group. The false positive score assesses the probability that genes considered significant based on a specific *P* value from the experimental group are a result of random chance as estimated by the scramble control group. False positive scores lower than 0.05 were considered significant.

#### Searching mouse phenotype database

To determine the functional relevance of the identified genes (Fig. 4C), the mouse phenotype database was downloaded from MGI (www.informatics.jax.org/phenotypes.shtml). Specifically, two files were used in this analysis: “MPheno_OBO.ontology,” which includes phenotype IDs and parental relations; and “MGI_PhenoGenoMP.rpt,” which includes all genotypes and phenotype annotations. Genes with any phenotype annotation in these files were used in this analysis. Genes annotated with any phenotype under hematopoietic system phenotype (ID: 0005397) or immune system phenotype (ID: 0005387) are considered to be associated with a hematopoietic/immune system phenotype.

#### Classifying quantitative association patterns

To classify the association patterns across various amounts of HSC lineage output, we plotted −log_10_( combined *P* value) against the rank of HSC lineage output, and fitted the data points to the following three formulas in order (Fig. 5A and fig. S8):

y=a (1)

$$y=b\times e^{-\frac{{\left(x-\mu \right)}^{2}}{2\sigma^{2}}}+c$$ (2)

$$y=\left\{\begin{array}{c} d,\text{if}\;x\leq f\\ e,\text{if}\;x>f \end{array}\right.$$ (3)

*a*, *b*, *c*, *d*, *e*, *f*, μ, and σ are constants calculated by the Python function “scipy.stats.curve_fit”. Genes whose data points best fit formula 1 were classified as a constant association. For the remaining genes, those whose data points best fit formula 2 were classified as the unimodal association. For the remaining genes, those whose data points best fit formula 3 were classified as the discrete association. Last, data from all the remaining genes were fitted to a polynomial formula using a Python function “numpy.polyfit,” applying the minimum degree when *r*^2^ of the fitting reaches 0.9. We then identified the number of peaks on the polynomial curves to classify the genes into either the unimodal group (one peak) or the multimodal group (multiple peaks).

#### Statistical analysis

Statistical analyses were performed using the Python package “scipy.stats.” Unpaired two-tailed Student’s *t* test was used to determine statistical significance in Fig. 1 (D and E). An unpaired two-tailed Wilcoxon rank-sum test was used for other statistical significance calculations. In all cases, *P* < 0.05 was considered significant.
