# Supplementary material for: Quantitative association between gene expression and blood cell production of individual hematopoietic stem cells in mice
Source: Sci Adv. 2024 Jan 26;10(4):eadk2132. doi: 10.1126/sciadv.adk2132 (PMC10816716; doi:10.1126/sciadv.adk2132)
Supplement: Supplementary file 1 — Figs. S1 to S13 Tables S1 to S4 [file sciadv.adk2132_sm.pdf]

Science Advances
AAAS

Supplementary Materials for

Quantitative association between gene expression and blood cell production of individual hematopoietic stem cells in mice

Du Jiang *et al.*

Corresponding author: Rong Lu, ronglu@usc.edu

Sci. Adv. **10**, eadk2132 (2024)
DOI: 10.1126/sciadv.adk2132

This PDF file includes:

Figs. S1 to S13
Tables S1 to S4

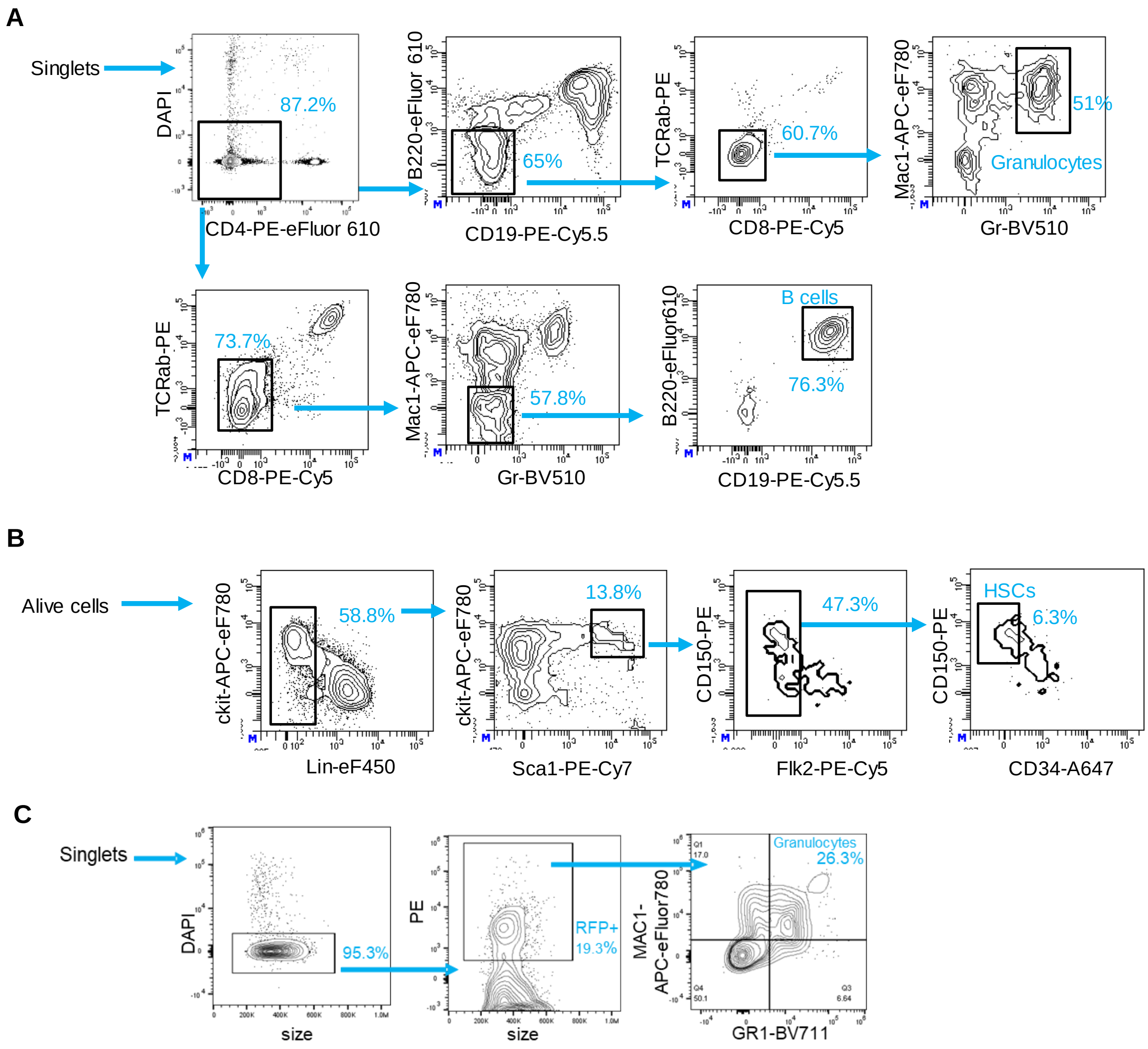

Fig. S1. FACS gating for cell isolation. (A) FACS gating for sorting granulocytes and B cells from the peripheral white blood cells. **(B)** FACS gating for sorting HSCs from c-kit enriched bone marrow cells. (**C**) FACS gating for in vitro differentiation assay analysis.

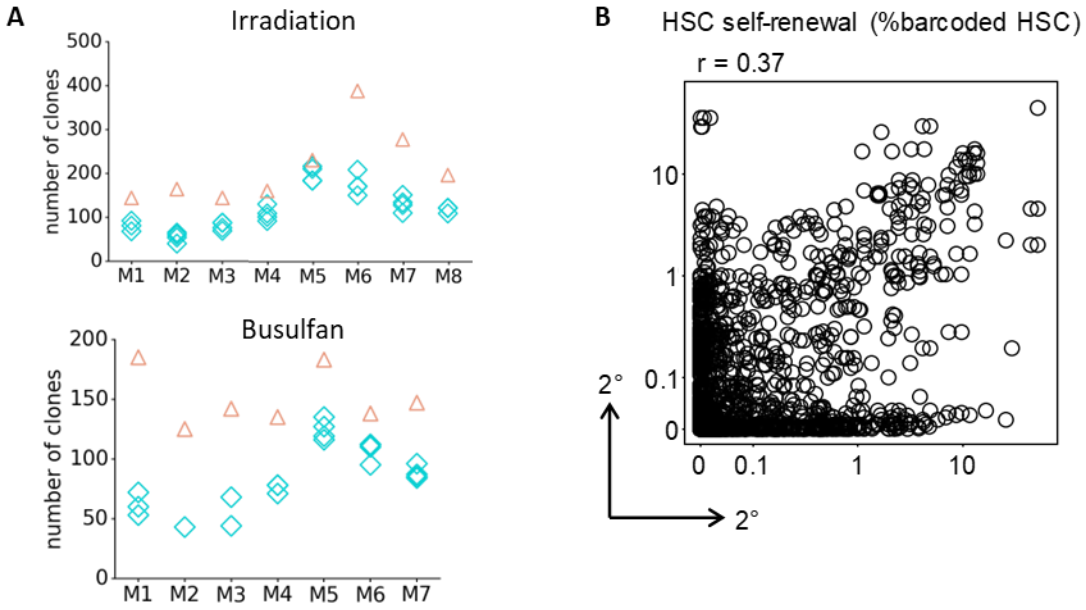

Fig. S2. Comparing the self-renewal of HSCs derived from the same ancestor. (A) Number of detected clones in each primary recipient (orange triangle) and its corresponding secondary recipients (blue diamonds) aligned vertically. (**B**) Shown are the abundances of HSC clones from secondary (2°) recipients that share a common primary recipient. Each open circle represents an HSC clone. Clones from eight secondary recipients are plotted. Shown are pairwise comparisons among all possible mouse pairs for each clone. "r" depicts Pearson correlation coefficient.

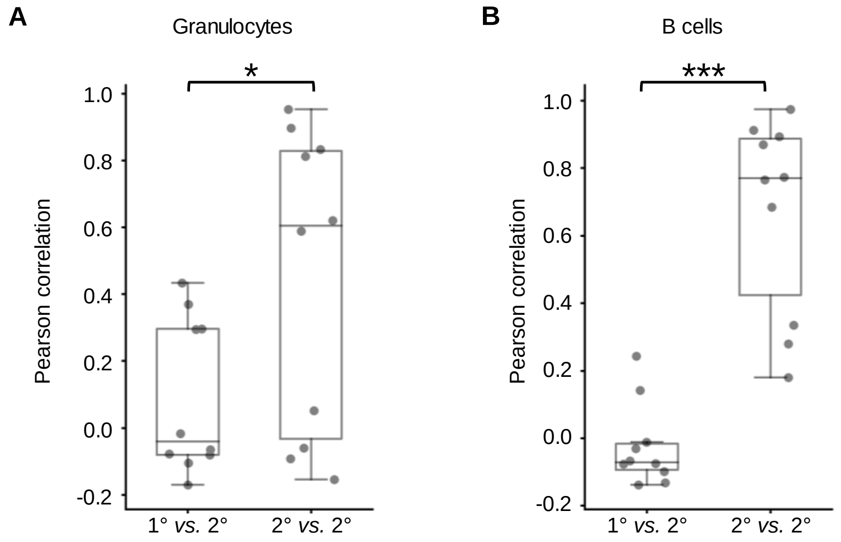

Fig. S3. One-to-multiple serial transplantation experiment using purified HSCs. Pearson correlation coefficient for the clonal abundance of granulocytes (**A**) or B cells (**B**) between one primary recipient and one secondary recipient (1° *vs.* 2°) and between two secondary recipients (2° *vs.* 2°). Each marker represents a comparison between a pair of mice. Experiments were performed similarly to those shown in Fig. 1, except that FACS-purified HSCs were used as donor cells for the secondary transplantation. * $P < 0.05$, *** $P < 0.001$.

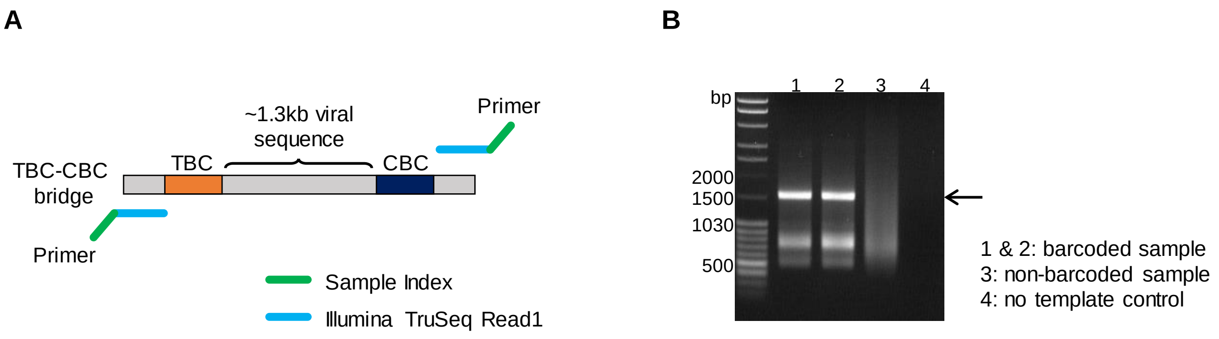

Fig. S4. Extracting "molecular bridges" containing both tracking barcodes and cell barcodes. (A) The PCR primers were designed to specifically amplify molecules that contain both a tracking barcode (TBC) and a cell barcode (CBC) from single cell cDNA libraries. **(B)** Agarose gel showing the PCR product. The arrow points to the desired products, the molecular bridges, that were cut and purified for PacBio sequencing.

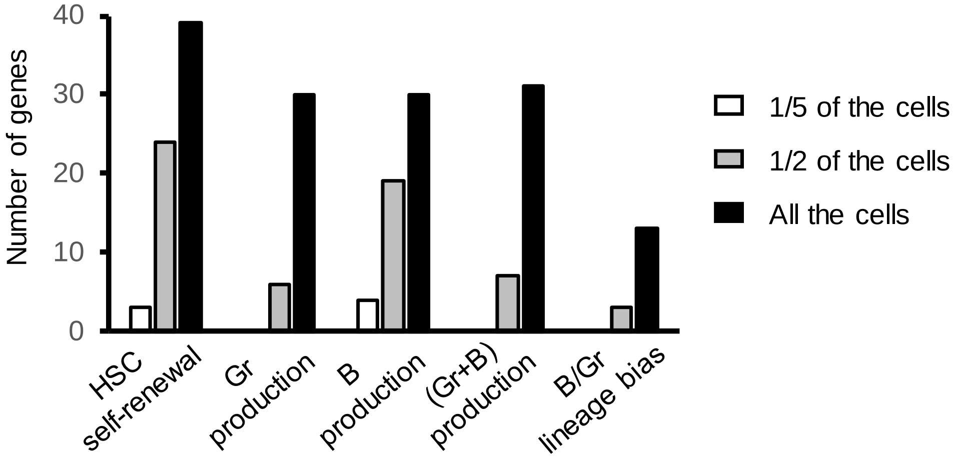

Fig. S5. Fewer genes were identified when data from fewer cells were used in the analyses. Shown are the number of genes that significantly associated with HSC lineage output at false positive scores (FPS) less than 0.05 using different number of cells and the algorithm outlined in Fig. 3A.

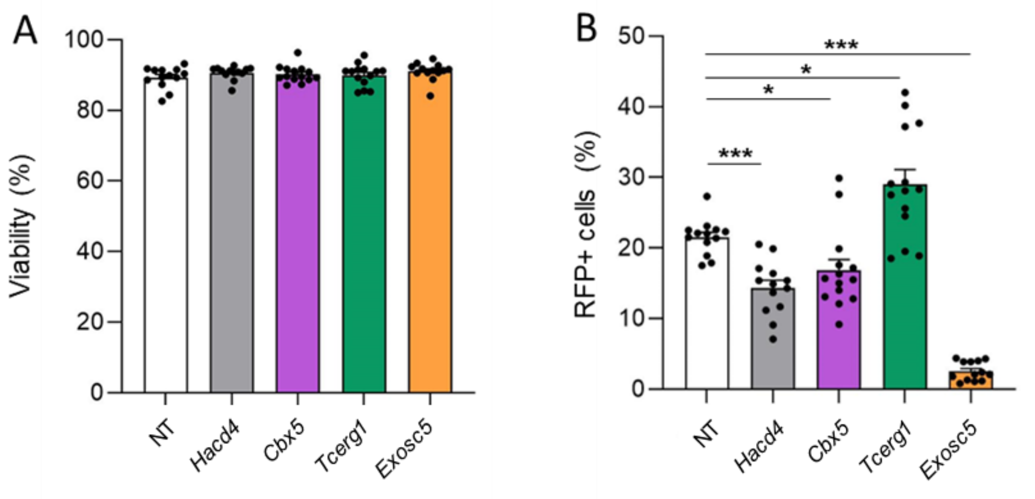

Fig. S6. *In vitro* functional assay of selected genes negatively associated with granulocyte production. Cas9 expressing HSCs were transduced with lentiviral vectors carrying a mix of 3 different sgRNAs that target *Cbx5*, *Tcerg1*, *Exosc5*, or *Hacd4* and NT, non-targeting sgRNAs. (A) Cell viability after 8 days of myeloid differentiation culture. (B) Fraction of cells successfully transduced with sgRNAs among all alive cells. The experiment was performed twice (n=13 total replicates, except for *Tcerg1* and *Cbx5* n=14). Each dot depicts data from 1 well. Data shown as mean ± SEM. Two-tailed Student's t-test. * $P < 0.05$; *** $P < 0.001$.

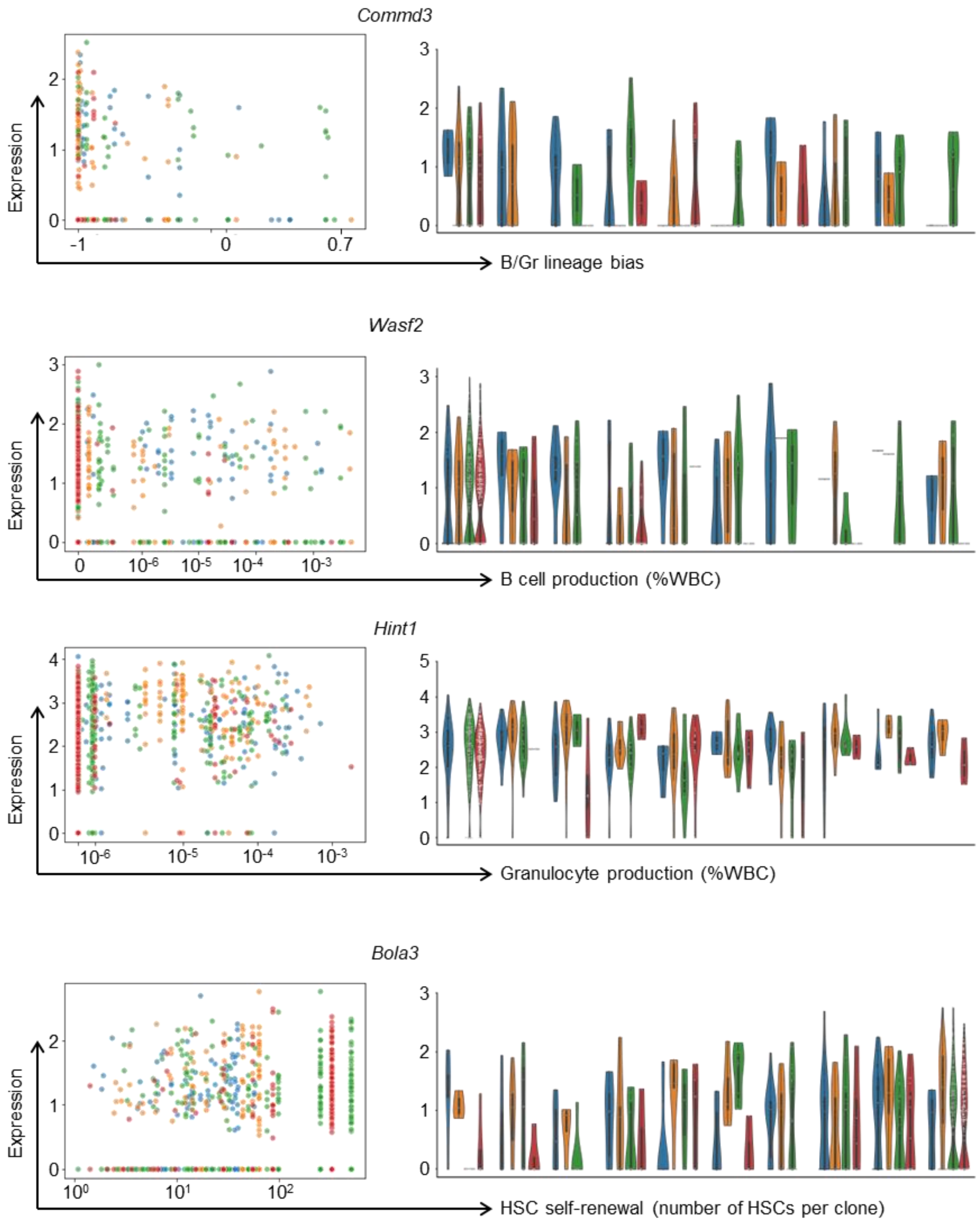

Fig. S7. Examples of quantitative comparison between gene expression and lineage output of individual HSCs. Shown are four example genes as in Fig. 5B. Each color represents data from one mouse. In the left panels, each dot represents data from one cell. Some dots overlap, particularly those with expression levels at 0. In the right panels, the lineage output values on the x-axis are split into ten equal bins, and the violin plot shows the distribution of the gene expression levels within each bin.

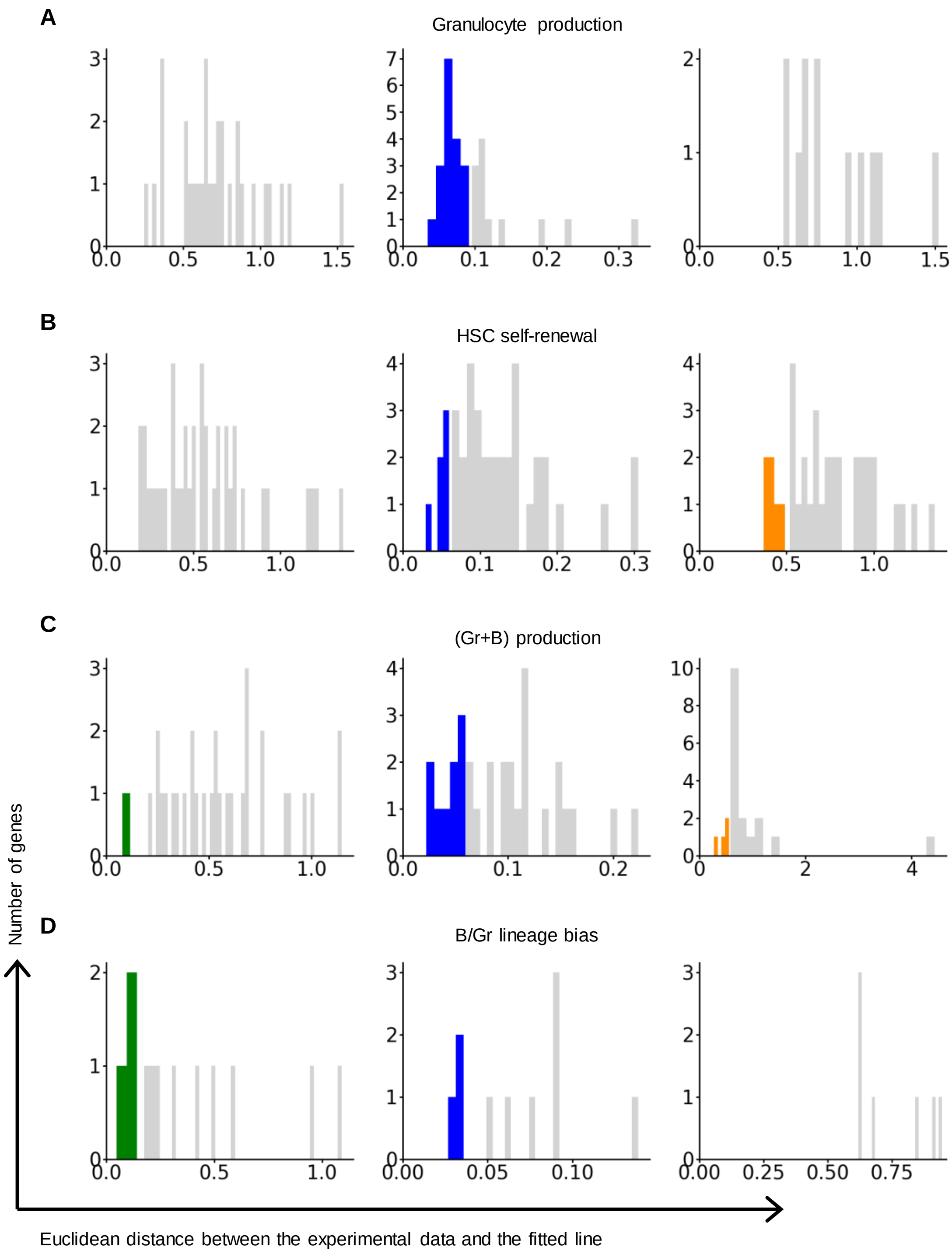

Fig. S8. Classification of quantitative association patterns. The Euclidean distance distribution and cutoff threshold for classifying genes that are significantly associated with granulocyte production (**A**), HSC self-renewal (**B**), total of granulocyte (Gr) and B cell production (**C**), and B cell / granulocyte lineage bias (**D**). See Fig. 5A for B cell production and more details.

A

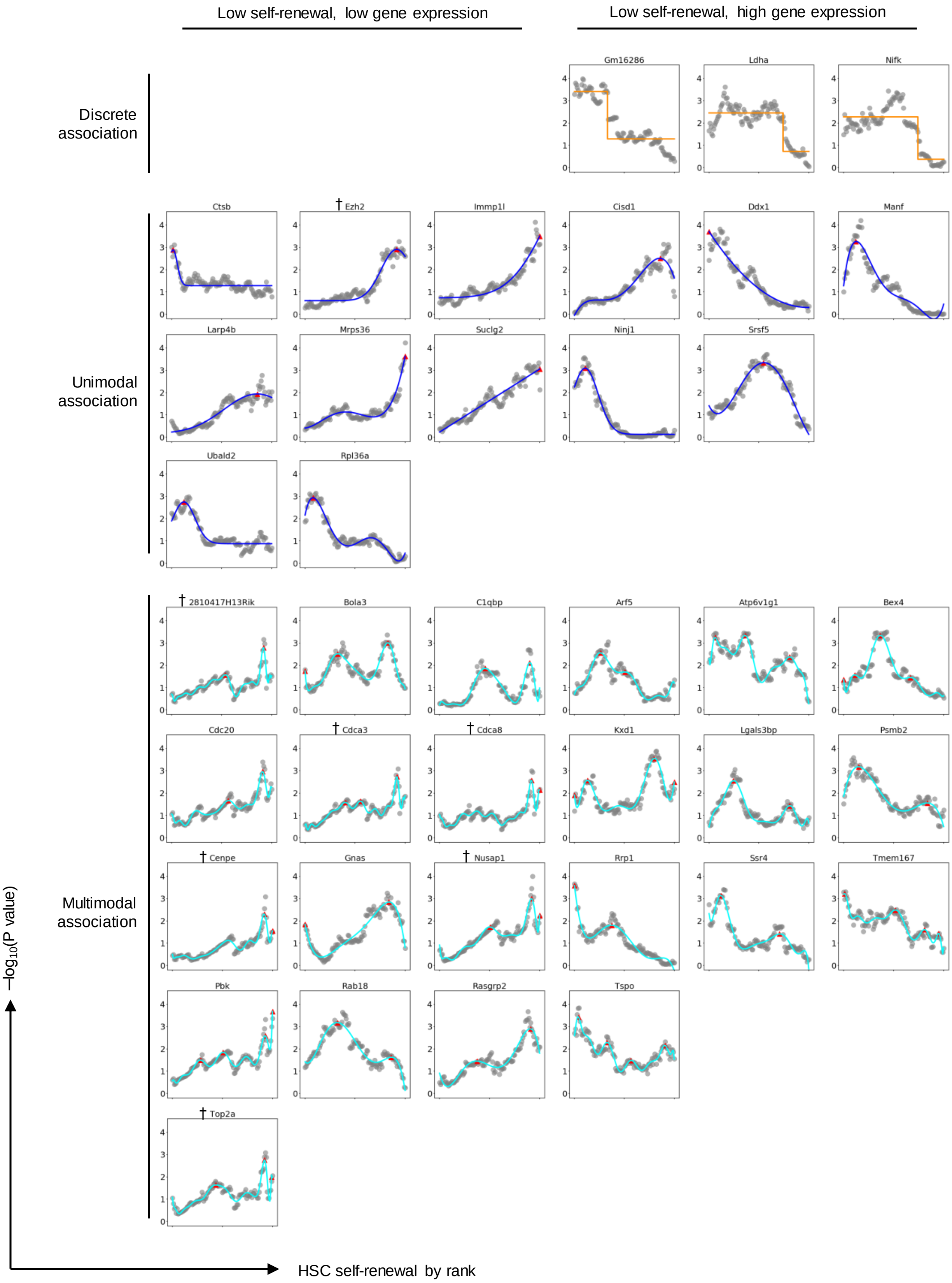

Low self-renewal, low gene expression
Low self-renewal, high gene expression
Discrete association
Unimodal association
Multimodal association
Gm16286
Ldha
Nifk
Ctsb
† Ezh2
Immp1l
Cisd1
Ddx1
Manf
Larp4b
Mrps36
Suclg2
Ninj1
Srsf5
Ubald2
Rpl36a
† 2810417H13Rik
Bola3
C1qbp
Arf5
Atp6v1g1
Bex4
Cdc20
† Cdca3
† Cdca8
Kxd1
Lgals3bp
Psmb2
† Cenpe
Gnas
† Nusap1
Rrp1
Ssr4
Tmem167
Pbk
Rab18
Rasgrp2
Tspo
† Top2a
−log10(P value)
HSC self-renewal by rank

B

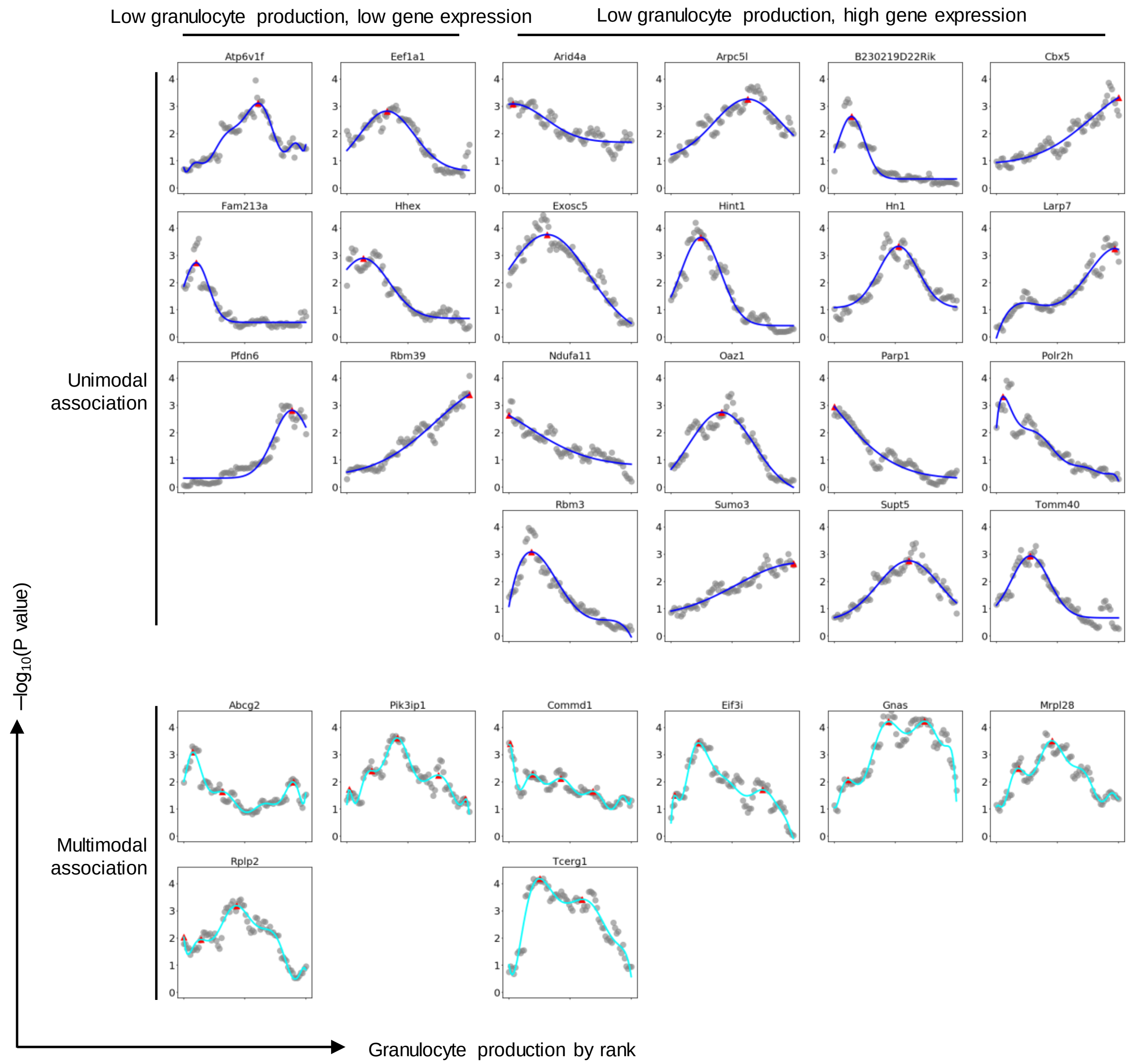
Low granulocyte production, low gene expression
Low granulocyte production, high gene expression
Unimodal association
Multimodal association
−log10(P value)
Granulocyte production by rank
Atp6v1f
Eef1a1
Arid4a
Arpc5l
B230219D22Rik
Cbx5
Fam213a
Hhex
Exosc5
Hint1
Hn1
Larp7
Pfdn6
Rbm39
Ndufa11
Oaz1
Parp1
Polr2h
Rbm3
Sumo3
Supt5
Tomm40
Abcg2
Pik3ip1
Commd1
Eif3i
Gnas
Mrpl28
Rplp2
Tcerg1

C

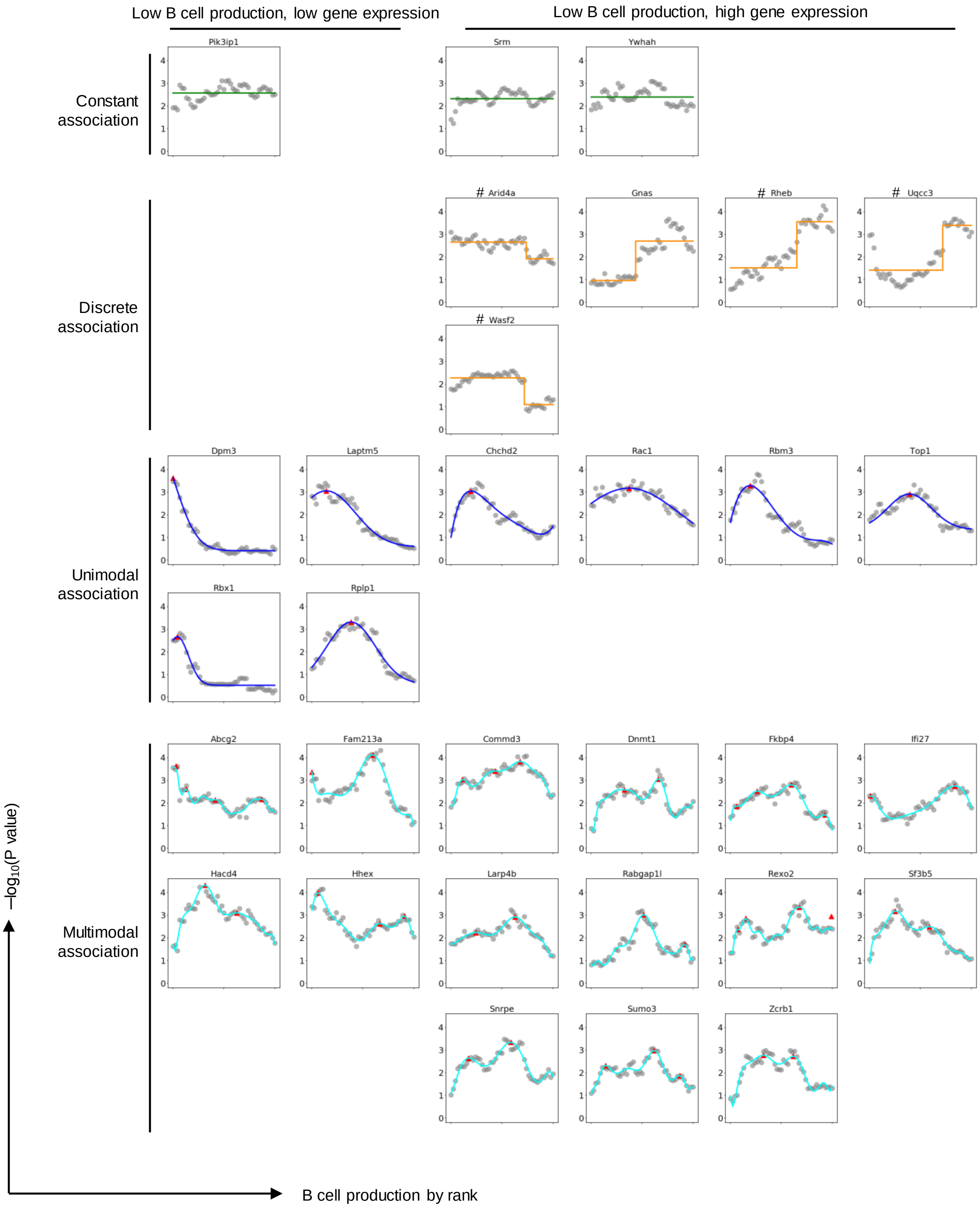

Low B cell production, low gene expression
Low B cell production, high gene expression
Constant association
Discrete association
Unimodal association
Multimodal association
Pik3ip1
Srm
Ywhah
Arid4a
Gnas
Rheb
Uqcc3
Wasf2
Dpm3
Laptm5
Chchd2
Rac1
Rbm3
Top1
Rbx1
Rplp1
Abcg2
Fam213a
Commd3
Dnmt1
Fkbp4
Ifi27
Hacd4
Hhex
Larp4b
Rabgap1l
Rexo2
Sf3b5
Snrpe
Sumo3
Zcrb1
$-\log_{10}$(P value)
B cell production by rank

D

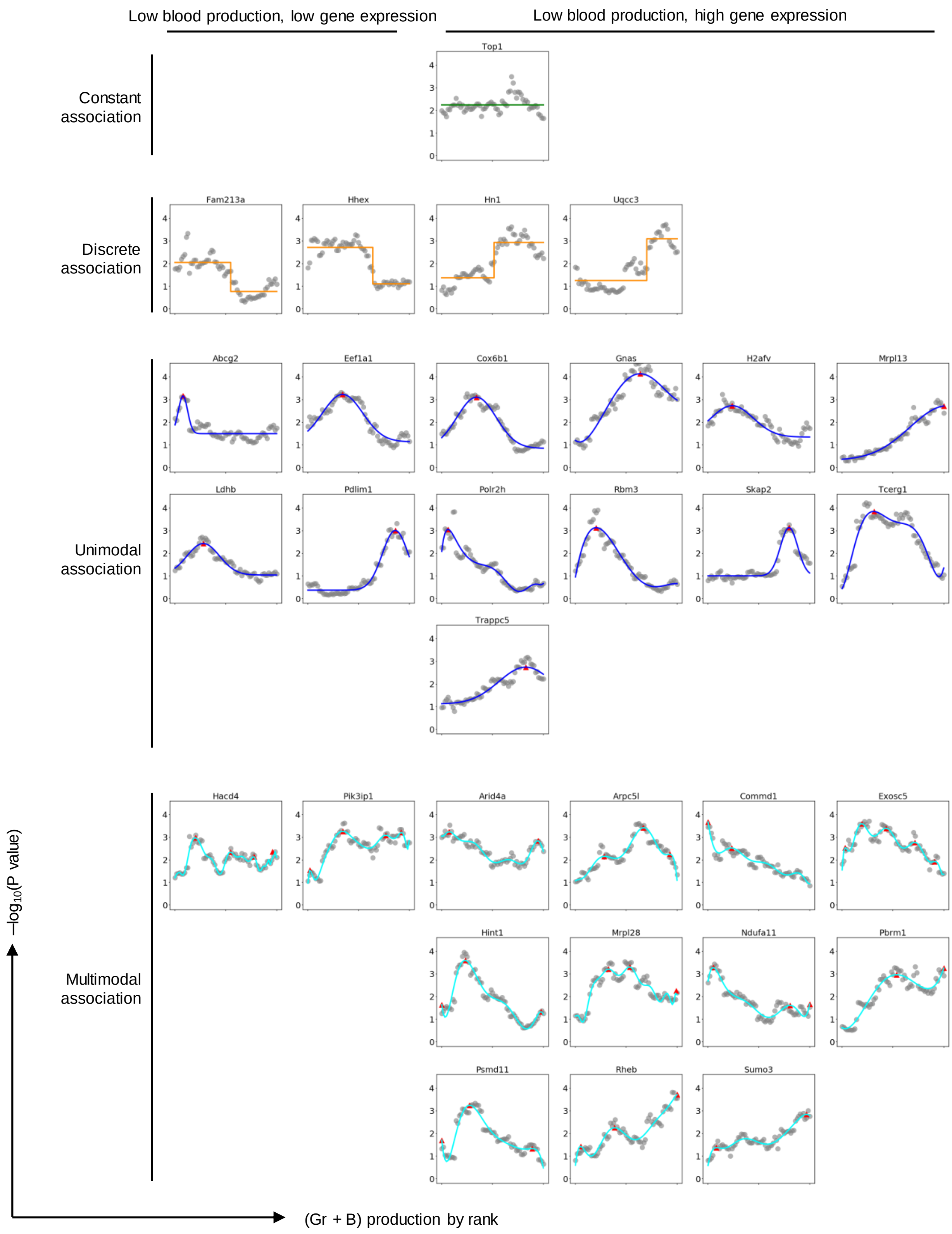

Low blood production, low gene expression
Low blood production, high gene expression
Constant association
Top1
Discrete association
Fam213a
Hhex
Hn1
Uqcc3
Unimodal association
Abcg2
Eef1a1
Cox6b1
Gnas
H2afv
Mrpl13
Ldhb
Pdlim1
Polr2h
Rbm3
Skap2
Tcerg1
Trappc5
Multimodal association
Hacd4
Pik3ip1
Arid4a
Arpc5l
Commd1
Exosc5
Hint1
Mrpl28
Ndufa11
Pbrm1
Psmd11
Rheb
Sumo3
−log$_{10}$(P value)
(Gr + B) production by rank

E

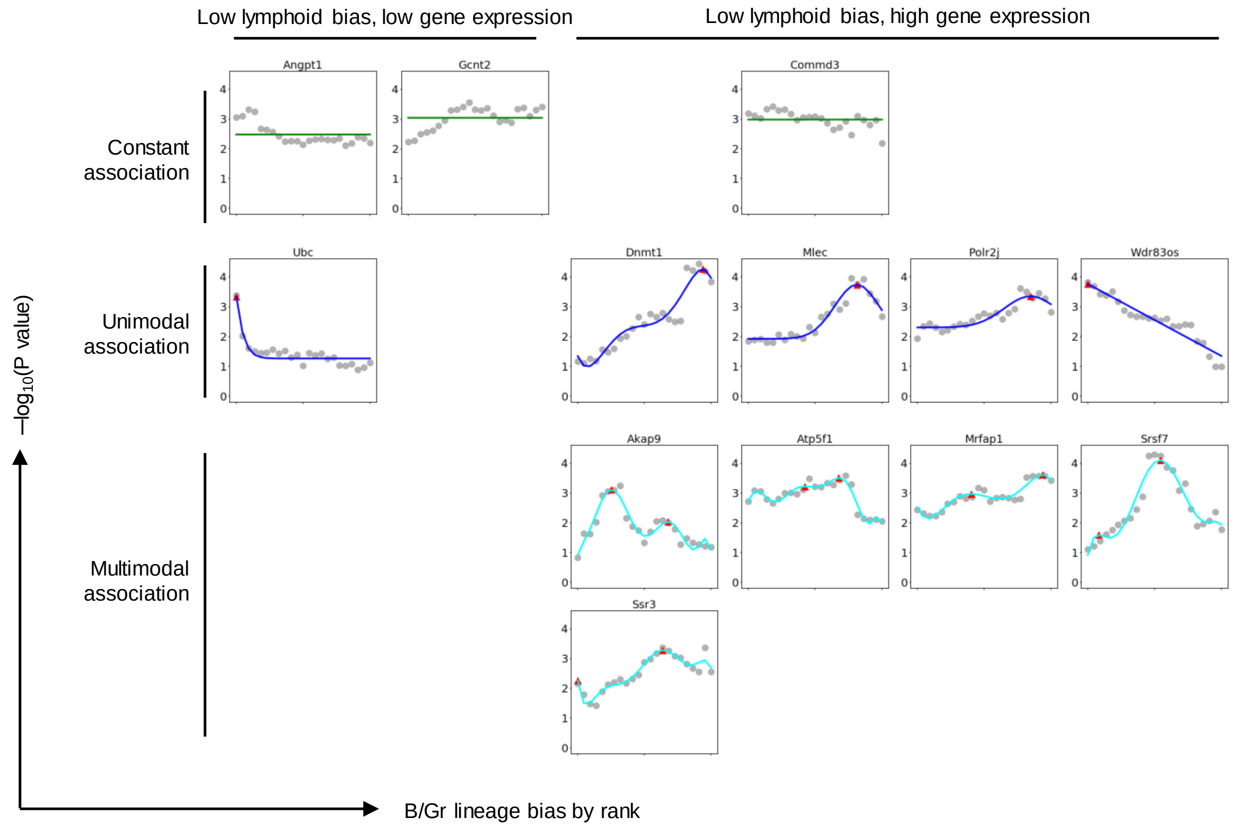

Fig. S9. Quantitative association patterns between lineage output and gene expression across individual HSCs. Shown are all genes identified as significantly associated with HSC self-renewal (**A**), granulocyte production (**B**), B cell production (**C**), total of granulocyte and B cell production (**D**), and B cell / granulocyte lineage bias (**E**). "†" in (A) denotes genes highlighted in Fig. 6B. "#" in (**C**) denotes genes highlighted in Fig. 6A.

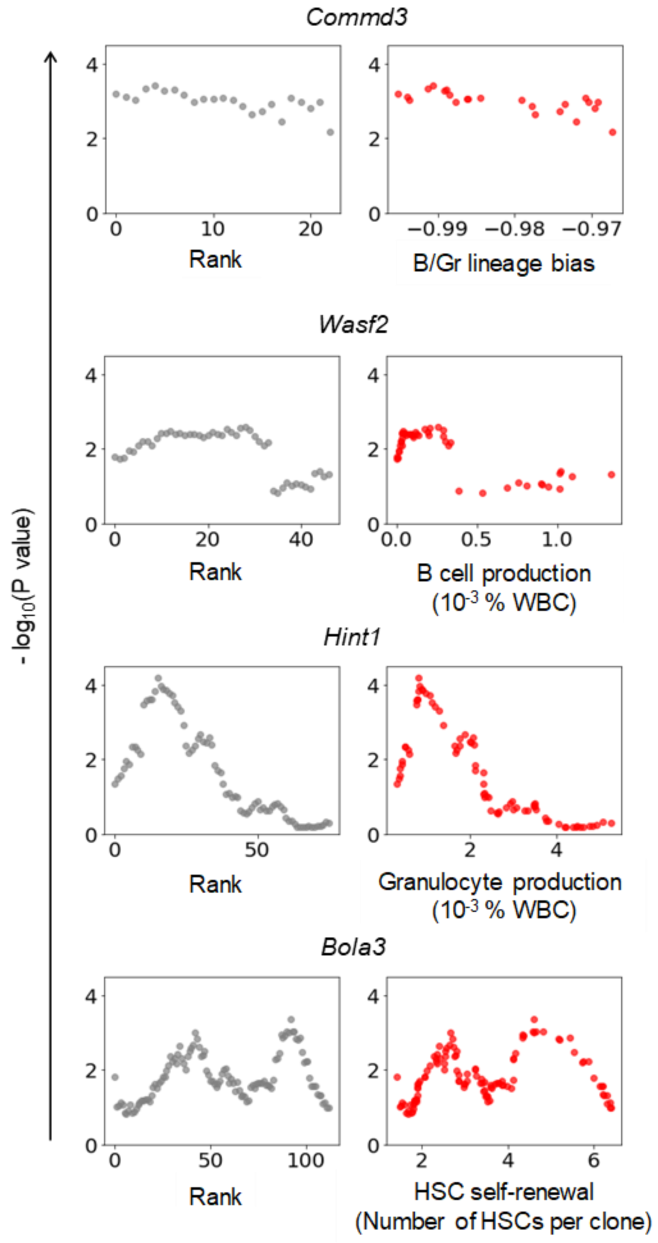

Fig. S10. Quantitative association patterns are not influenced if absolute values of HSC lineage output are used instead of rank. Shown are example patterns as those in Fig. 5B. Each grey or red dot represents a P-value calculated using one detected HSC lineage output value as the threshold.

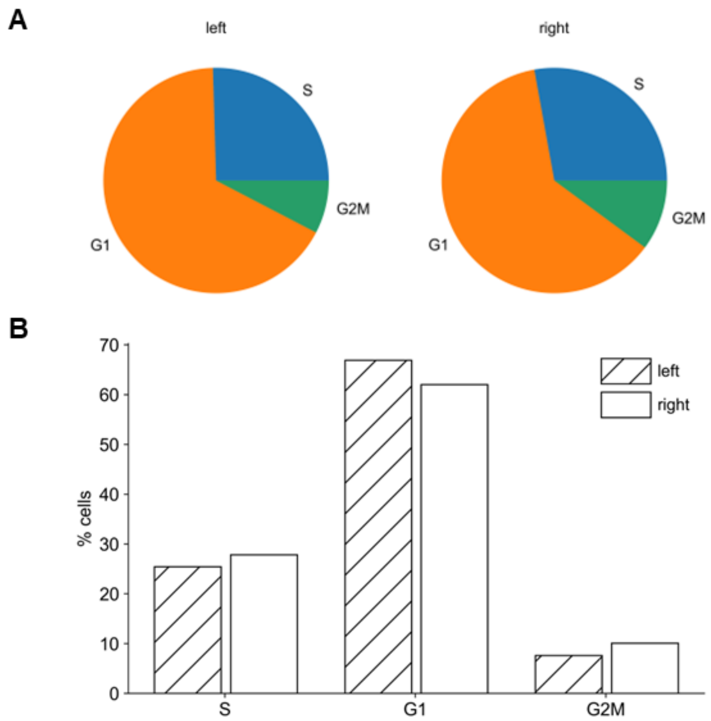

Fig. S11. Cell cycle distribution of HSCs with high or low levels of self-renewal. (**A**) Pie chart and (**B**) bar plot showing the cell cycle distribution of HSCs that are on the left or right side of the yellow highlighted line in Fig. 6B, which corresponds to HSCs with low or high levels of self-renewal, respectively.

Motif #1

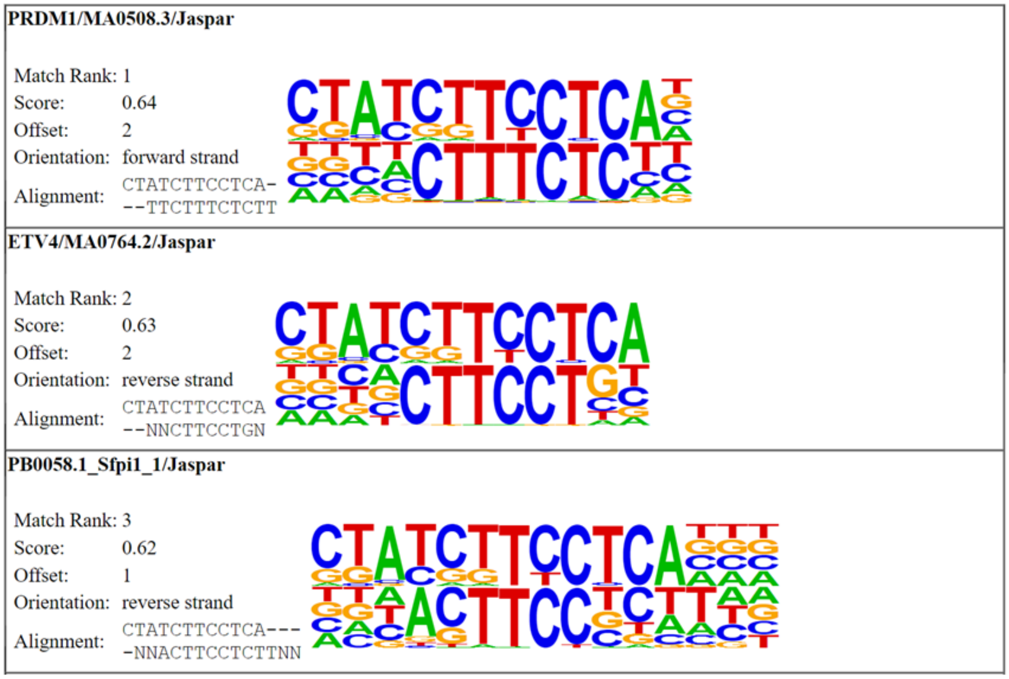

Motif #2

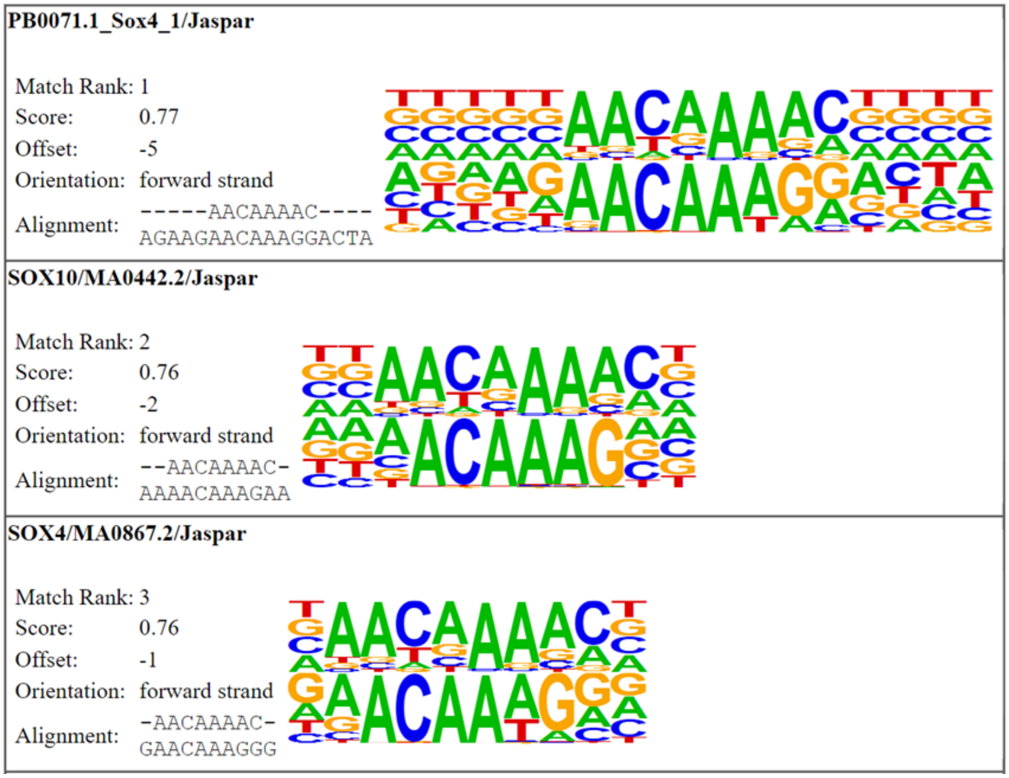

Fig. S12. Transcription factors with the most similar binding motifs as those in Fig. 7A. Shown are top three best matched transcription factors in mice.

Cenpe

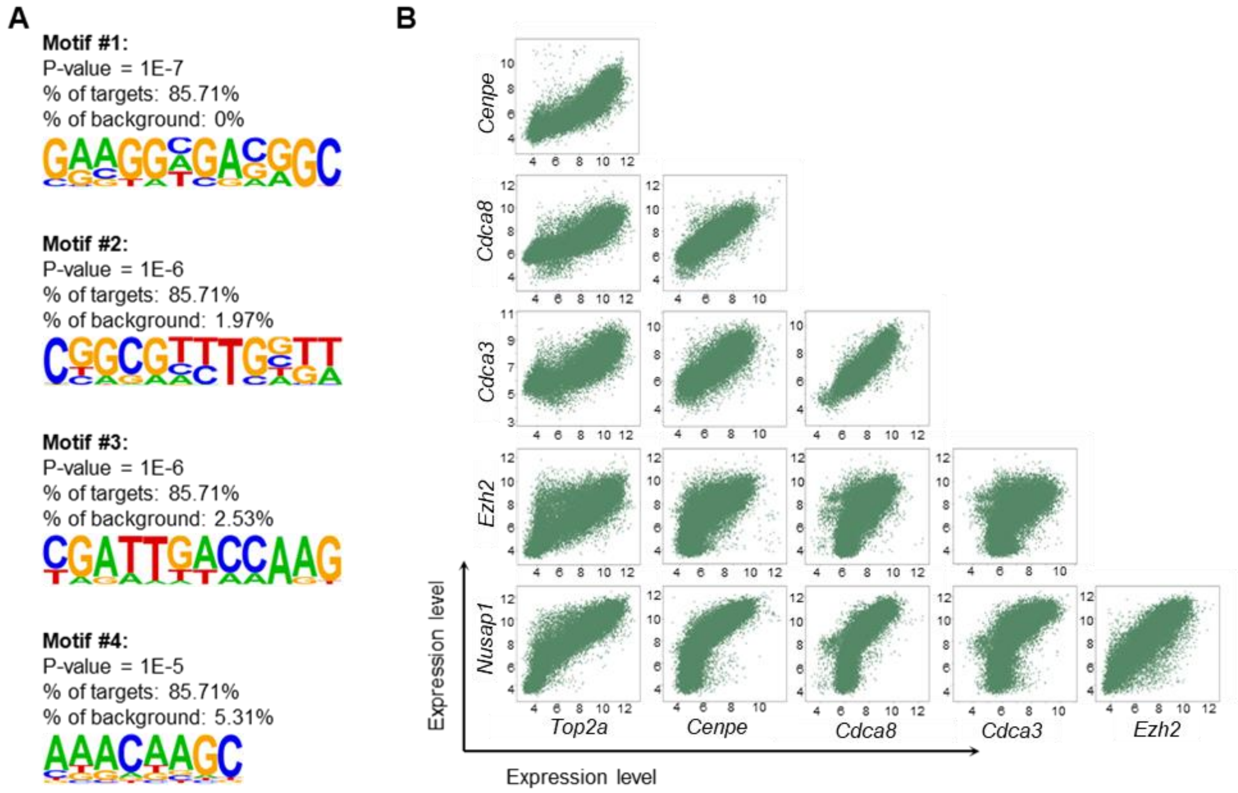

Fig. S13. Analyzing human homologs of genes with overlapping association peaks highlighted in yellow in Fig. 6B. (A) Common motif analysis was performed similarly as in Fig. 7A. The human homologs of the background genes in Fig. 7A were used as background. **(B)** Pairwise comparison of the transcription levels as measured by mRNA microarrays across various human tissues. Analysis was performed similarly as in Fig. 7C (http://hegemon.ucsd.edu/Tools/explore.php?key=global, Dataset "Human U133 Plus 2.0"). One of the seven highlighted genes (2810417H13Rik) was not found in the dataset. Each dot represents data from one microarray analysis. The position of the dot shows the normalized expression levels of the corresponding genes.

Table S1.

Examples of previous studies that showed the relevant functions of the genes that we identified as associated with HSC self-renewal or granulocyte production.

Abbreviations: AML – acute myeloid leukemia; BM – bone marrow; CML – chronic myeloid leukemia; GSCs – glioma stem-like cells; MDS – myelodysplastic syndrome; MLL – mixed-lineage leukemia; OSCC - oral squamous cell carcinoma; PB – peripheral blood.

**Gene**	**Our analysis**	**Previous study (experimental model and result summary)**	**References**
Ctsb	Higher expression in HSCs associated with higher self-renewal	• Pediatric AML and healthy PB and BM, glioma-initiating cells<br><br>• Higher expression and activity in AML patients<br>• Cysteine cathepsins play a role in HSCs adhesion in BM, trafficking and maturation<br>• Essential for maintaining stemness of glioma-initiating cells	Pandey G. et al., "Prognostic and therapeutic relevance of cathepsin B in pediatric acute myeloid leukemia." *Am J Cancer Res*. 9, no. 12 (2019):2634-2649 *(40)*<br><br>Arora M. at al., "Cysteine Cathepsins and Their Prognostic and Therapeutic Relevance in Leukemia." *Ann Natl Acad Med Sci* 57, no. 2 (2021):108-116 *(41)*<br><br>Sreelatha G. et al., "Cathepsin B and uPAR regulate self-renewal of glioma-initiating cells through GLI-regulated Sox2 and Bmi1 expression." *Carcinogenesis*, 34, no. 3 (2013): 550–559 *(42)*
Ezh2	Higher expression in HSCs associated with higher self-renewal	• Mouse HSCs, transplantation<br><br>• Overexpression in HSCs prevented loss of stem cells quality in serial transplantation	Kamminga L.M. at al., "The Polycomb group gene *Ezh2* prevents hematopoietic stem cell exhaustion." *Blood* 107, no. 5 (2006): 2170–2179 *(43)*
Larp4b	Higher expression in HSCs associated with higher self-renewal	• Knockdown MLL-AF9 AML mouse model<br><br>• Knockdown decreased leukemia cells in PB, BM and spleen<br>• Knockdown impaired the self-renewal of leukemia stem cells	Zhang Y. et al., "La-related protein 4B maintains murine MLL-AF9 leukemia stem cell self-renewal by regulating cell cycle progression." *Exp Hematol*. 43, no. 4 (2015): 309-18.e2 *(44)*

Cdc20	Higher expression in HSCs associated with higher self-renewal	• Human glioma stem-like cells, cell cultures, and xenotransplantation<br>• Cdc20 is crucial for in vivo tumorigenicity of GSCs	Mao D.D. et al., “CDC20-APC/SOX2 Signaling Axis Regulates Human Glioblastoma Stem-like Cells.” *Cell Reports* 11, no. 11 (2015): 1809-1821 *(45)*
Gnas	Higher expression in HSCs associated with higher self-renewal	• Mouse bone marrow cell transplantation<br>• Ectopic expression of $GNAS^{R201C}$ (gain-of-function missense mutation) supported transplantable HSC activity	Ostrander E.L. et al., “The GNAS R201C mutation associated with clonal hematopoiesis supports transplantable hematopoietic stem cell activity.” *Exp Hematol* 57 (2018): 14-20 *(46)*
Nusap1	Higher expression in HSCs associated with higher self-renewal	• Hepatocellular carcinoma xenograft mouse models and cell lines; human cervical cancer cells lines<br>• Overexpression increased while knockdown decreased the number of liver cancer stem cells<br>• Overexpression increased spheroid formation<br>• Upregulation promoted self-renewal of cervical cancer cells (tumor sphere formation)	Li J. et al., “NUSAP1, a novel stemness-related protein, promotes early recurrence of hepatocellular carcinoma.” *Cancer Sci.* 113, (2022): 4165- 4180 *(47)*<br><br>Li, H., et al.” Nucleolar and spindle associated protein 1 promotes metastasis of cervical carcinoma cells by activating Wnt/β-catenin signaling.” *J Exp Clin Cancer Res* 38, (2019):33 *(48)*
Pbk	Higher expression in HSCs associated with higher self-renewal	• Transgenic mice and neuronal progenitor cells culture<br>• PBK/TOPK-positive cells are mitotically active progenitors in vivo<br>• Inhibition of PBK/TOPK and P38 pathway disrupts progenitor proliferation and self-renewal	Dougherty J.D. et al., “PBK/TOPK, a Proliferating Neural Progenitor-Specific Mitogen-Activated Protein Kinase Kinase.” *J Neurosci* 25, no. 46 (2005): 10773-10785 *(49)*

Bex4	Higher expression in HSCs associated with lower self-renewal	• Oral squamous cell carcinoma cell lines<br><br>• Overexpression reduced while silencing increased OSCC proliferation	Gao W. et al., "Decreased brain-expressed X-linked 4 (BEX4) expression promotes growth of oral squamous cell carcinoma." J Exp Clin Cancer Res 35, (2016): 92 *(50)*
Hhex	Higher expression in HSCs associated with higher granulocyte and B cell production	• Knockout mouse model and cultures, transplantation, human AML<br><br>• Overexpressed in AML<br>• Promoted myeloid progenitor expansion and AML leukemogenesis	Shields B.J. et al., "Acute myeloid leukemia requires Hhex to enable PRC2-mediated epigenetic repression of Cdkn2a." *Genes Dev*. 30, no. 1 (2016):78-91 *(51)*<br><br>Jackson J.T. et al., "Hhex regulates murine lymphoid progenitor survival independently of Stat5 and Cdkn2a." *Eur. J. Immunol.*, 50 (2020): 959-971 *(52)*<br><br>Jackson, J.T. et al. "A crucial role for the homeodomain transcription factor Hhex in lymphopoiesis." *Blood* 125, no. 5 (2015): 803-814. *(53)*
Rbm39	Higher expression in HSCs associated with higher granulocyte production	• Human AML PB or BM and normal human CD34+ HSPCs, human and mouse leukemia cell culture, transplantation<br><br>• Higher expression in AML patients' samples<br>• Required to sustain leukemia survival in vivo and in vitro<br>• Drug-induced RBM39 degradation exerted anti-leukemic effects	Wang, E. et al., "Targeting an RNA-binding protein network in acute myeloid leukemia." *Cancer Cell* 35, no. 3 (2019): 369-384.e7 *(54)*<br><br>Hsiehchen, D. et al., "Biomarkers for RBM39 degradation in acute myeloid leukemia." *Leukemia* 34, (2020): 1924–1928 *(55)*
Abcg2	Higher expression in HSCs associated with higher granulocyte production	• Mouse bone marrow cell transplantation, human MDS samples<br><br>• Overexpression drives myelodysplastic syndrome<br>• Expressed at significantly higher levels in	Kawabata, K.C. et al., "High expression of ABCG2 induced by EZH2 disruption has pivotal roles in MDS pathogenesis." *Leukemia* 32, no. 2 (2018): 419. *(56)*

		myelodysplastic syndrome patients	
Arid4a	Higher expression in HSCs associated with lower granulocyte production	• Knockout mouse model<br><br>• Knockout mouse developed acute myeloid leukemia	Wu, M. et al., "Identification of chromatin remodeling genes Arid4a and Arid4b as leukemia suppressor genes." *Journal of the National Cancer Institute* 100, no. 17 (2008): 1247-1259. *(57)*
Hint1	Higher expression in HSCs associated with lower granulocyte production	• Knockout mouse models, human A-AML and euploid €-AML cases<br>• Knockout led to increased tumor incidence in aged mice<br>• Knockout embryo fibroblasts had enhanced growth rate and were resistant to radiation-induced cytotoxicity<br>• Decreased expression in E-AML as compared to A-AML	Li, H., et al. "*Hint1* is a haplo-insufficient tumor suppressor in mice." *Oncogene* 25, (2006): 713–721 *(58)*<br><br>Su T. et al., "Deletion of histidine triad nucleotide-binding protein 1/PKC-interacting protein in mice enhances cell growth and carcinogenesis." Proc Natl Acad Sci USA 100 no. 13 (2003):7824-9 *(59)*<br><br>Simonetti G. et al., "Aneuploid acute myeloid leukemia exhibits a signature of genomic alterations in the cell cycle and protein degradation machinery." *Cancer* 125, no. 5 (2019):712-725 *(60)*
Oaz1	Higher expression in HSCs associated with lower granulocyte production	• CML patient samples and cell line, human oral cancer cell line<br><br>• Downregulated in CML patients<br>• Overexpression elevated cell erythroid differentiation and apoptosis<br>• Overexpression inhibited cell proliferation of oral cancer cells	Wu B. et al., "Assay of OAZ1 mRNA Levels in Chronic Myeloid Leukemia Combined with Application of Leukemia PCR Array Identified Relevant Gene Changes Affected by Antizyme." *Acta Hematol* 131, (2014):141-147 *(61)*<br><br>Wang X. and Jiang l., "Effects of ornithine decarboxylase antizyme 1 on the proliferation and differentiation of human oral cancer cells." *Int J Mol Med* 34, (2014): 1606-1612 *(62)*
Parp1	Higher expression in HSCs associated with lower	• MDS patients BM primary cells culture<br><br>• Olaparib, a potent PARP1 inhibitor, increased the	Faraoni I. et al., "Cytotoxicity and Differentiating Effect of the Poly(ADP-Ribose) Polymerase Inhibitor Olaparib in Myelodysplastic Syndromes." *Cancers* 11, no. 9 (2019):1373 *(63)*

	granulocyte production	number of metamyelocytes and mature granulocytes<br>• PARP1 suppression follows HSCs differentiation to monocytes and repression of GATA2 and RUNX1	Sobczak, M. et al., "The Role of PARP1 in Monocyte and Macrophage Commitment and Specification: Future Perspectives and Limitations for the Treatment of Monocyte and Macrophage Relevant Diseases with PARP Inhibitors" *Cells* 9, no. 9 (2020): 2040. *(64)*
Sumo3	Higher expression in HSCs associated with lower granulocyte production	• Zebrafish transgenic lines, transgenic mice, various cell lines<br><br>• Deficiency favors myelopoiesis at the expense of erythropoiesis during primitive hematopoiesis in transgenic zebrafish<br>• Loss of SUMO2/3 binding, enhanced the myeloid transformational potential of c-Myb<br>• SUMO-deficient (K160R) PML/RARA induces myeloid hyperplasia in mice	Hao Y. et al., “Sumoylation of CCAAT/enhancer–binding protein α promotes the biased primitive hematopoiesis of zebrafish.” *Blood* 117 no. 26 (2011): 7014–7020 *(65)*<br><br>Zhu, J. et al., “A sumoylation site in PML/RARA is essential for leukemic transformation.” *Cancer Cell* 7, (2005): 143–153 *(66)*<br><br>Kukkula, A. et al., “Therapeutic Potential of Targeting the SUMO Pathway in Cancer.” *Cancers* 13, (2021): 4402. *(67)*

Table S2.
A complete list of genes exhibiting significant associations with lineage output of individual HSCs.

HSC lineage output	Association pattern	Genes	
		Low lineage output, low gene expression	Low lineage output, high gene expression
HSC self-renewal	Discrete		*Gm16286, Ldha, Nifk*
	Unimodal	*Ctsb, Ezh2, Immp1l, Larp4b, Mrps36, Suclg2, Ubald2, Rpl36a*	*Cisd1, Ddx1, Manf, Ninj1, Srsf5*
	Multimodal	*2810417H13Rik, Bola3, C1qbp, Cdc20, Cdca3, Cdca8, Cenpe, Gnas, Nusap1, Pbk, Rab18, Rasgrp2, Top2a*	*Arf5, Atp6v1g1, Bex4, Kxd1, Lgals3bp, Psmb2, Rrp1, Ssr4, Tmem167, Tspo*
Granulocyte production	Unimodal	*Atp6v1f, Eef1a1, Fam213a, Hhex, Pfdn6, Rbm39*	*Arid4a, Arpc5l, B230219D22Rik, Cbx5, Exosc5, Hint1, Hn1, Larp7, Ndufa11, Oaz1, Parp1, Polr2h, Rbm3, Sumo3, Supt5, Tomm40*
	Multimodal	*Abcg2, Pik3ip1, Rplp2*	*Commd1, Eif3i, Gnas, Mrpl28, Tcerg1*
B cell production	Constant	*Pik3ip1*	*Srm, Ywhah*
	Discrete		*Arid4a, Gnas, Rheb, Uqcc3, Wasf2*
	Unimodal	*Dpm3, Laptm5, Rbx1, Rplp1*	*Chchd2, Rac1, Rbm3, Top1*
	Multimodal	*Abcg2, Fam213a, Hacd4, Hhex*	*Commd3, Dnmt1, Fkbp4, Ifi27, Larp4b, Rabgap1l, Rexo2, Sf3b5, Snrpe, Sumo3, Zcrb1*
(Gr+B) production	Constant		*Top1*
	Discrete	*Fam213a, Hhex*	*Hn1, Uqcc3*
	Unimodal	*Abcg2, Eef1a1, Ldhb, Pdlim1*	*Cox6b1, Gnas, H2afv, Mrpl13, Polr2h, Rbm3, Skap2, Tcerg1, Trappc5*
	Multimodal	*Hacd4, Pik3ip1*	*Arid4a, Arpc5l, Commd1, Exosc5, Hint1, Mrpl28,*

			Ndufa11, Pbrm1, Psmd11, Rheb, Sumo3
B/Gr lineage bias	Constant	*Angpt1, Gcnt2*	*Commd3*
	Unimodal	*Ubc*	*Dnmt1, Mlec, Polr2j, Wdr83os*
	Multimodal		*Akap9, Atp5f1, Mrfap1, Srsf7, Ssr3*

Table S3. sgRNA sequences and Synthego's ICE analysis results

Gene	sgRNA sequence	Indel %	KO score
Tcerg1	GGTGGGAACATCATACCCGG	52	43
	TAGAACACTAGAATCCACGT		
	GGTAGATGTCGGAGTTGACG		
Hacd4	TATTTGCCAGTATCCATGAG	53	46
	GGTGATCACCAGTCAAGAGG		
	AAACTTACCTGCAAAAACCG		
Exosc5	CCCCCGGACCTCAATCACCG	69	35
	TGGCCGAGAAGAGCCGCGAG		
	CAGGAATACCTGTGAAGCTG		
Cbx5	GGATGAGGAGGAATATGTGG	47	48
	AAGTGGAATATCTGTTGAAG		
	AGAAATCAGAAGGAAACAAG		

Table S4. Select Gene Ontology annotations of the genes in Fig. 6B.

Gene	Gene Ontology Annotations
Top2a	DNA topoisomerase type II activity, ATP binding, chromosome, hematopoietic progenitor cell differentiation, histone deacetylase binding, female meiosis chromosome separation.
Cenpe	ATP binding, attachment of spindle microtubules to kinetochore, chromosome, mitotic cell cycle, positive regulation of protein kinase activity, cell division, cytoplasm.
Cdca8	chromosome passenger complex, cell cycle, cell division, cytoplasm, mitotic sister chromatid segregation, spindle midzone.
Cdca3	molecular function, cytoplasm, adherent junction, cell cycle, cell division.
Nusap1	establishment of mitotic spindle localization, microtubule binding, mitotic chromosome condensation, cell cycle, cell division, positive regulation of mitotic nuclear division.
Ezh2	histone H3K27 methylation, B cell differentiation, positive regulation of cell cycle G1/S phase transition, chromatin organization, positive regulation of GTPase activity.
2810417H13Rik	chromatin binding, DNA replication, centrosome cycle, cytoplasm, regulation of cell cycle, cellular response to DNA damage stimulus.
